# Gene × Physical Activity Interactions in Obesity: Combined Analysis of 111,421 Individuals of European Ancestry

**DOI:** 10.1371/journal.pgen.1003607

**Published:** 2013-07-25

**Authors:** Shafqat Ahmad, Gull Rukh, Tibor V. Varga, Ashfaq Ali, Azra Kurbasic, Dmitry Shungin, Ulrika Ericson, Robert W. Koivula, Audrey Y. Chu, Lynda M. Rose, Andrea Ganna, Qibin Qi, Alena Stančáková, Camilla H. Sandholt, Cathy E. Elks, Gary Curhan, Majken K. Jensen, Rulla M. Tamimi, Kristine H. Allin, Torben Jørgensen, Soren Brage, Claudia Langenberg, Mette Aadahl, Niels Grarup, Allan Linneberg, Guillaume Paré, InterAct Consortium, DIRECT Consortium, Patrik K. E. Magnusson, Nancy L. Pedersen, Michael Boehnke, Anders Hamsten, Karen L. Mohlke, Louis T. Pasquale, Oluf Pedersen, Robert A. Scott, Paul M. Ridker, Erik Ingelsson, Markku Laakso, Torben Hansen, Lu Qi, Nicholas J. Wareham, Daniel I. Chasman, Göran Hallmans, Frank B. Hu, Frida Renström, Marju Orho-Melander, Paul W. Franks

**Affiliations:** 1 Genetic and Molecular Epidemiology Unit, Lund University Diabetes Centre, Department of Clinical Sciences, Skåne University Hospital, Lund University, Malmö, Sweden; 2 Diabetes and Cardiovascular Disease - Genetic Epidemiology, Department of Clinical Sciences, Skåne University Hospital, Lund University, Malmö, Sweden; 3 Diabetes Epidemiology Research Group, Steno Diabetes Center, Gentofte, Denmark; 4 Department of Public Health and Clinical Medicine, Section for Medicine, Umeå University, Umeå, Sweden; 5 Department of Odontology, Umeå University, Umeå, Sweden; 6 Division of Preventive Medicine, Brigham and Women's Hospital and Harvard Medical School, Boston, Massachusetts, United States of America; 7 Department of Medical Epidemiology and Biostatistics, Karolinska Institutet, Stockholm, Sweden; 8 Department of Nutrition, Harvard School of Public Health, Boston, Massachusetts, United States of America; 9 Department of Medicine, University of Eastern Finland, Kuopio, Finland; 10 The Novo Nordisk Foundation Center for Basic Metabolic Research, Section of Metabolic Genetics, Faculty of Health and Medical Sciences, University of Copenhagen, Copenhagen, Denmark; 11 MRC Epidemiology Unit, Institute of Metabolic Science, Addenbrooke's Hospital, Cambridge, United Kingdom; 12 Department of Epidemiology, Harvard School of Public Health, Boston, Massachusetts, United States of America; 13 Channing Division of Network Medicine, Department of Medicine, Brigham and Women's Hospital and Harvard Medical School, Boston, Massachusetts, United States of America; 14 Research Centre for Prevention and Health, Glostrup University Hospital, Glostrup, Denmark; 15 Department of Pathology and Molecular Medicine, McMaster University, Hamilton, Ontario, Canada; 16 Genetic and Molecular Epidemiology Laboratory, McMaster University, Hamilton, Ontario, Canada; 17 Population Health Research Institute, Hamilton, Ontario, Canada; 18 Department of Public Health and Clinical medicine, Section for Nutritional Research, Umeå University, Umeå, Sweden; 19 Faculty of Health and Medical Sciences, University of Copenhagen, Copenhagen, Denmark; 20 Faculty of Health Sciences, University of Southern Denmark, Odense, Denmark; 21 Kuopio University Hospital, Kuopio, Finland; 22 Department of Biostatistics and Center for Statistical Genetics, University of Michigan School of Public Health, Ann Arbor, Michigan, United States of America; 23 Cardiovascular Genetics and Genomics Group, Atherosclerosis Research Unit, Department of Medicine, Solna, Karolinska Institutet, Stockholm, Sweden; 24 Department of Genetics, University of North Carolina, Chapel Hill, North Carolina, United States of America; 25 Department of Ophthalmology, the Mass Eye and Ear Infirmary, Harvard Medical School, Boston, Massachusetts, United States of America; 26 Faculty of Health Sciences, University of Aarhus, Aarhus, Denmark; 27 Division of Cardiology, Brigham and Women's Hospital and Harvard Medical School, Boston, Massachusetts, United States of America; 28 Department of Medical Sciences, Molecular Epidemiology and Science for Life Laboratory, Uppsala University, Uppsala, Sweden; 29 Division of Genetics, Brigham and Women's Hospital and Harvard Medical School, Boston, Massachusetts, United States of America; University of Alabama at Birmingham, United States of America

## Abstract

Numerous obesity loci have been identified using genome-wide association studies. A UK study indicated that physical activity may attenuate the cumulative effect of 12 of these loci, but replication studies are lacking. Therefore, we tested whether the aggregate effect of these loci is diminished in adults of European ancestry reporting high levels of physical activity. Twelve obesity-susceptibility loci were genotyped or imputed in 111,421 participants. A genetic risk score (GRS) was calculated by summing the BMI-associated alleles of each genetic variant. Physical activity was assessed using self-administered questionnaires. Multiplicative interactions between the GRS and physical activity on BMI were tested in linear and logistic regression models in each cohort, with adjustment for age, age^2^, sex, study center (for multicenter studies), and the marginal terms for physical activity and the GRS. These results were combined using meta-analysis weighted by cohort sample size. The meta-analysis yielded a statistically significant GRS × physical activity interaction effect estimate (*P_interaction_* = 0.015). However, a statistically significant interaction effect was only apparent in North American cohorts (n = 39,810, *P_interaction_* = 0.014 vs. n = 71,611, *P_interaction_* = 0.275 for Europeans). In secondary analyses, both the *FTO* rs1121980 (*P_interaction_* = 0.003) and the *SEC16B* rs10913469 (*P_interaction_* = 0.025) variants showed evidence of SNP × physical activity interactions. This meta-analysis of 111,421 individuals provides further support for an interaction between physical activity and a GRS in obesity disposition, although these findings hinge on the inclusion of cohorts from North America, indicating that these results are either population-specific or non-causal.

## Author Summary

We undertook analyses in 111,421 adults of European descent to examine whether physical activity diminishes the genetic risk of obesity predisposed by 12 single nucleotide polymorphisms, as previously reported in a study of 20,000 UK adults (Li *et al*, PLoS Med. 2010). Although the study by Li *et al* is widely cited, the original report has not been replicated to our knowledge. Therefore, we sought to confirm or refute the original study's findings in a combined analysis of 111,421 adults. Our analyses yielded a statistically significant interaction effect (*P_interaction_* = 0.015), confirming the original study's results; we also identified an interaction between the *FTO* locus and physical activity (*P_interaction_* = 0.003), verifying previous analyses (Kilpelainen *et al*, PLoS Med., 2010), and we detected a novel interaction between the *SEC16B* locus and physical activity (*P_interaction_* = 0.025). We also examined the power constraints of interaction analyses, thereby demonstrating that sources of within- and between-study heterogeneity and the manner in which data are treated can inhibit the detection of interaction effects in meta-analyses that combine many cohorts with varying characteristics. This suggests that combining many small studies that have measured environmental exposures differently may be relatively inefficient for the detection of gene × environment interactions.

## Introduction

Obesity is a major risk factor for many non-communicable diseases including type 2 diabetes, cardiovascular disease, and certain cancers [1]. Genetic predisposition and lifestyle factors are known to increase obesity susceptibility, and the technological breakthroughs that came with genome-wide association studies (GWAS) have led to the successful identification of a large number of obesogenic loci [2]–[6]. Recent studies suggest that physical activity may modify genetic susceptibility to obesity, with the genetic burden being higher in physically inactive compared with active persons [7]–[9]. The most extensively studied example of a gene × physical activity interaction in obesity is for the *FTO* locus [7], [10], which was recently replicated in a meta-analysis comprising 240,000 persons [11]. Elsewhere, Li *et al* reported that physical activity offsets the aggregated genetic risk of 12 obesogenic loci [12].

In the current study, we aimed to replicate the findings of Li *et al* [12] in a sample collection of 111,421 individuals of European ancestry. We also undertook detailed analyses focused on the role of within- and between-study factors to establish how the design of gene × environment interaction meta-analyses impacts the power to detect interactions.

## Results

Supplementary Table S1 shows participant characteristics for each of the 11 participating cohorts.

### Genetic risk score (GRS) × physical activity interactions

**Figure 1:**
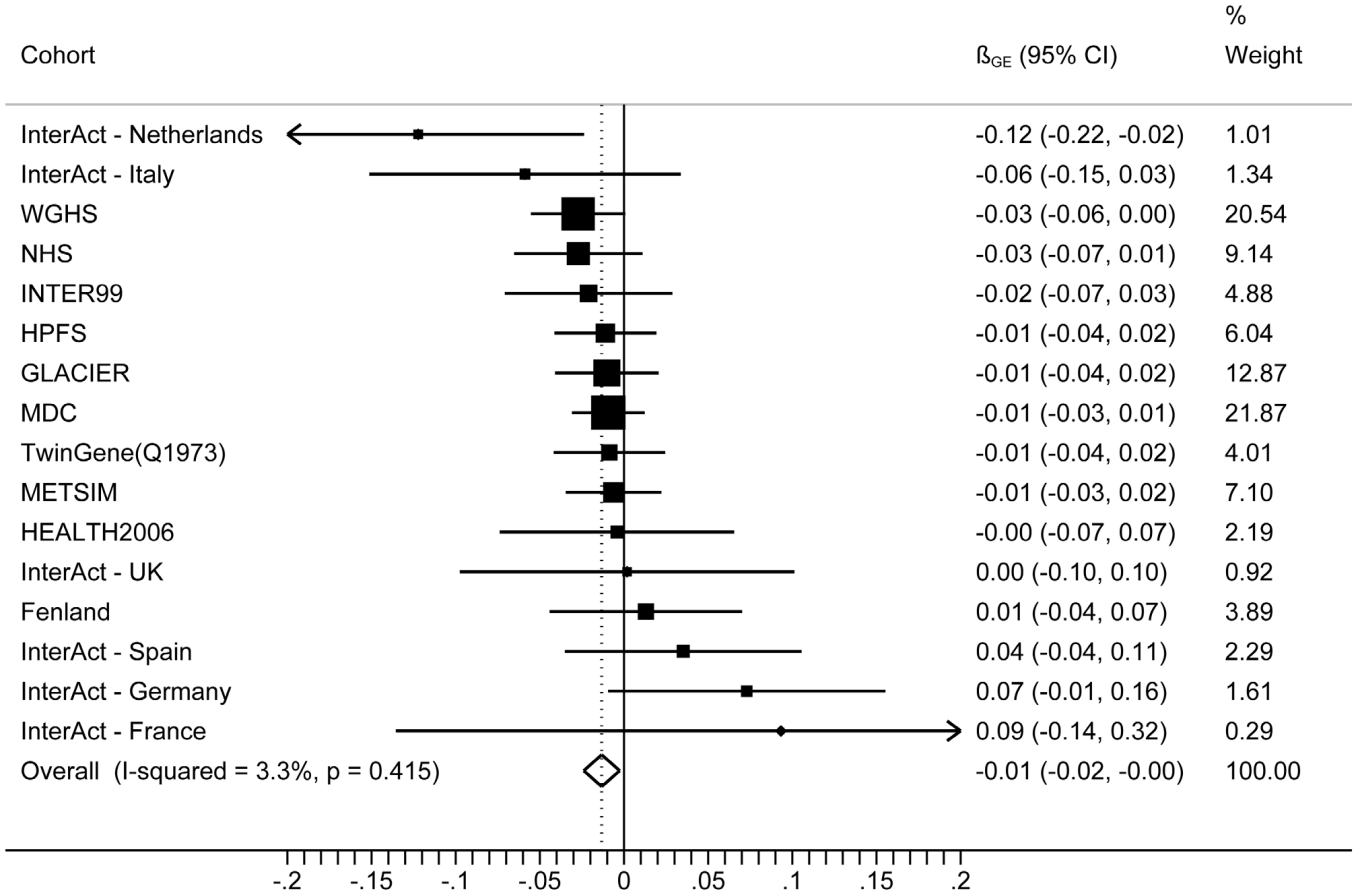
Forest plot showing the meta-analysis of interaction coefficients (GRS × Cambridge Physical Activity Index) in relation to BMI (11 cohorts; N = 111,421) (*P_interaction_* = 0.015).

**Figure 2:**
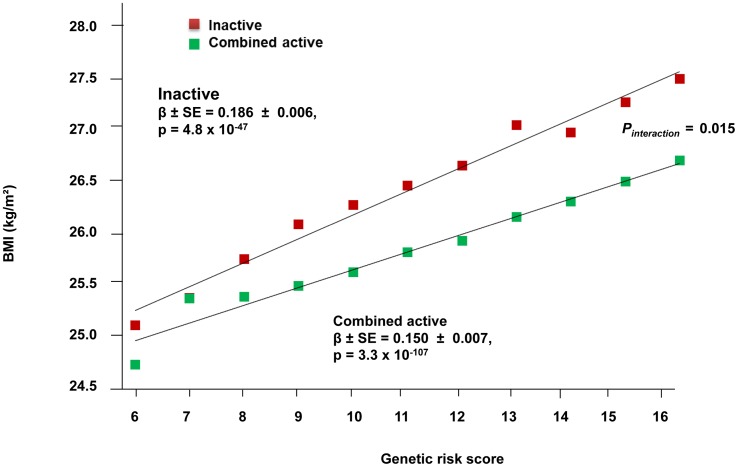
Association between the GRS and BMI in the inactive and ‘combined active’ groups (N = 111,421). Physical activity was estimated according to the Cambridge Physical Activity Index (CPAI), where the inactive group is defined as individuals with a CPAI of 1 and the ‘combined active’ group as individuals with a CPAI of 2–4.

**Figure 3:**
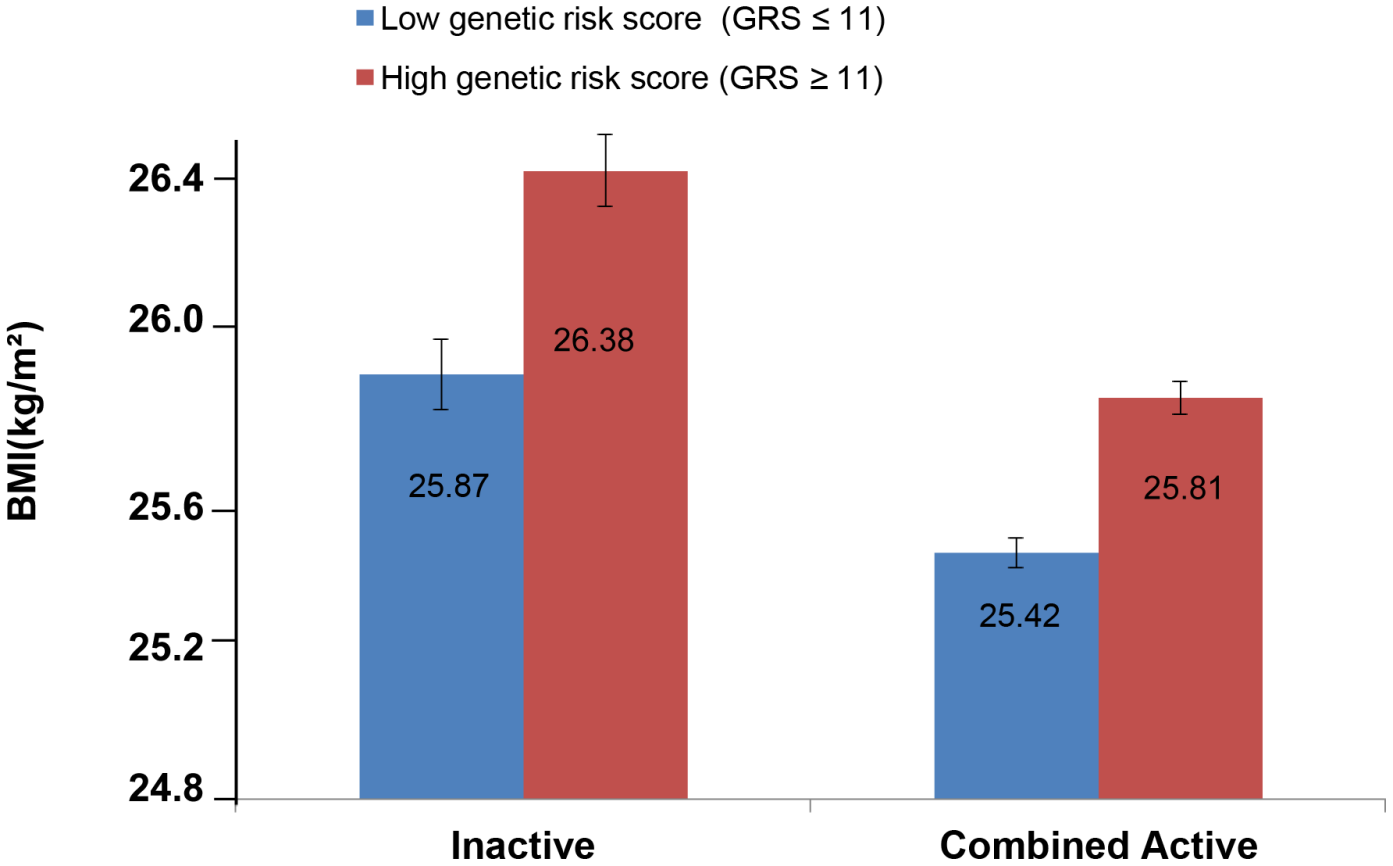
Adjusted least square mean BMI (95% CI) stratified by GRS level (>11 vs. ≤11 BMI-associated alleles) and by physical activity levels (N = 111,421). Physical activity was estimated according to the Cambridge Physical Activity Index (CPAI), where the ‘inactive’ group is defined as individuals with CPAI = 1 and the ‘combined active’ group as individuals with CPAI = 2–4.

The forest plot in Figure 1 shows the interaction coefficients across the 11 cohorts included in the meta-analysis, along with the overall interaction effect estimate (*P_interaction_* = 0.015). Table 1 summarizes the adjusted main effects of the GRS on BMI and obesity in the combined data from all cohorts and by strata of physical activity. Each unit increase in the GRS, equivalent to one BMI-raising allele, was associated with a mean 0.161 (SE = 0.006) kg/m^2^ higher BMI (*P* = 2.1×10^−176^), which corresponds to 465 g heavier weight for a person 1.70 m tall. Overall, among physically inactive individuals (with a Cambridge Physical Activity Index [CPAI] of 1), each additional BMI-raising allele was associated with 0.186 (SE = 0.006) kg/m^2^ higher BMI, equivalent to 538 g in weight for a person 1.70 m tall (*P* = 4.8×10^−47^), whereas the effect in the most physically active group (CPAI of 4) was 0.143 kg/m^2^ per GRS allele (SE = 0.011, *P* = 5.6×10^−40^), or 413 g in weight for a person 1.70 m tall. In the ‘combined active’ group (individuals with a CPAI of 2–4), each additional risk allele was associated with 0.150 kg/m^2^ (SE = 0.007, *P* =  3.3×10^−107^) higher BMI, or 434 g in weight for a person 1.70 m tall (Figure 2). As illustrated in Figure 3, in the inactive group (CPAI of 1), the difference in BMI between persons with a low (≤11 alleles) and high (>11 alleles) GRS was 0.647 kg/m^2^ (SE = 0.06; *P* = 1.9×10^−25^), while the difference in the combined active group was 0.532 kg/m^2^ (SE = 0.03; *P* = 6.6×10^−67^).

**Table 1:** Association of the genetic risk score with BMI and risk of obesity adjusted for age, age^2^, and sex in the combined sample of all 11 cohorts and further stratified by physical activity level.

Physical activity level^a^	N	β^b^ (SE)	*P*-value	β_weight_ c	n (normal weight)/n (obese)	OR^d^ (95% CI)	*P*-value
Overall	111,421	0.161 (0.006)	2.1×10^−176^	465	52,714/16,506	1.081 (1.069, 1.094)	1.1×10^−42^
Inactive	27,847	0.186 (0.006)	4.8×10^−47^	538	11,451/5,696	1.090 (1.072, 1.107)	2.3×10^−25^
Moderately inactive	31,956	0.160 (0.011)	3.8×10^−51^	462	14,978/4,695	1.052 (1.031, 1.075)	1.6×10^−06^
Moderately active	27,440	0.155 (0.011)	1.1×10^−46^	478	13,859/3,441	1.093 (1.073, 1.114)	8.5×10^−21^
Active	24,178	0.143 (0.011)	5.6×10^−40^	413	10,945/4,155	1.095 (1.071, 1.120)	1.7×10^−15^

a Physical activity was estimated according to the Cambridge Physical Activity Index (CPAI), which categorizes total physical activity levels on a four level scale.

b Increase in BMI units (kg/m^2^) for each additional unit increase in the GRS (equivalent to one additional risk allele).

c β converted to body weight (g) for a person 1.70 m tall.

d higher odds of being obese (≥30 kg/m^2^) versus normal weight (18.5≤BMI<25 kg/m^2^) for each additional BMI-increasing allele.

The CPAI characterizes total physical activity levels by considering both occupational and leisure time physical activity [13]. Sensitivity analyses were performed in the GLACIER and MDC cohorts (n = 39,000) where interaction terms (gene × physical activity) were modeled separately for occupational and leisure time physical activity, but these results were not materially different from the main analyses (data not shown). Within these two cohorts, we additionally adjusted the models for putative confounding by smoking and education, but the results were essentially the same irrespective of whether these additional covariates were or were not included; hence, for the sake of comparability, we focus on the results with the regression models adjusted as reported by Li *et al* [12]. We also undertook sensitivity analyses in European and North American cohorts separately (Supplementary Figures S1a and S1b), which revealed a statistically significant GRS × physical activity interaction effect in the latter (n = 39,810, *P_interaction_* = 0.014), but not the former (n = 71,611, *P_interaction_* = 0.275).

### Individual SNP × physical activity interactions

In analyses modeling the interaction of each of the 12 individual SNPs and physical activity, two tests of interaction were nominally statistically significant: the *FTO* rs1121980 variant, which concurs with previous reports of interaction at this locus [11], and the *SEC16B* rs10913469 locus, which has not previously been reported (Table 2). It should be noted that several of the cohorts used here are included in Kilpeläinen *et al.* [11], and so this is not entirely independent confirmation of these findings. The magnitude of the interaction effects (β_GE_) for *FTO* rs1121980 and *SEC16B* rs10913469 variants was −0.052 and −0.049 kg/m^2^ per risk allele respectively, which compares with β_GE_ of −0.108 kg/m^2^ per 8.33 alleles for the GRS (equivalent to 1 allele on the bi-allelic scale). For *FTO*, the interaction effect was almost 10-fold larger in North American than in European cohorts, whereas for the *SEC16B* locus, the interaction effect was approximately twice the magnitude in North American vs. European cohorts. Supplementary Table S2 shows individual SNP interaction results across each of the 11 cohorts. In models excluding the *FTO* and *SEC16B* variants from the GRS, the interaction test was no longer statistically significant (in the entire cohort [*P_interaction_* = 0.25] or separately within the cohorts from North American [*P_interaction_* = 0.39] and Europe [*P_interaction_* = 0.44]), strongly suggesting that the GRS × physical activity interaction result is driven by the inclusion of one or both of these variants.

**Table 2:** Meta-analyzed single SNP interactions with physical activity* on BMI.

SNPs	Nearest gene	β_GE_	(95% CI)	*P*_interaction_
rs1121980*	*FTO*	−0.052	(−0.086, −0.018)	0.003
rs7498665*	*SH2B1*	−0.003	(−0.039, 0.033)	0.867
rs10913469*	*SEC16B*	−0.049	(−0.091, −0.006)	0.025
rs10838738*	*MTCH2*	−0.012	(−0.047, 0.023)	0.502
rs17782313*	*MC4R*	−0.029	(−0.069, 0.010)	0.147
rs3101336*	*NEGR1*	0.006	(−0.028, 0.040)	0.728
rs6548238*	*TMEM18*	0.002	(−0.043, 0.047)	0.936
rs10938397	*GNPDA2*	−0.001	(−0.036,0.034)	0.946
rs925946*	*BDNF*	−0.013	(−0.052, 0.025)	0.491
rs368794*	*KCTD15*	−0.001	(−0.037, 0.035)	0.969
rs7647305*	*ETV5*	0.024	(−0.018, 0.066)	0.267
rs7132908*	*FAIM2*	−0.024	(−0.059, 0.010)	0.164

Physical activity was expressed according to the Cambridge Physical Activity Index (CPAI) (4 level scale); further details for the construction of the CPAI can be found in the *Materials and Methods* section and Table S7.

* Some studies used proxies for these variants, as reported in Table S8.

### Statistical power simulations

#### Power to detect interactions

We began by estimating power to detect the original interaction effect reported by Li *et al* [12] (Supplementary Figure S2a). We estimated that a sample size of N = 110,000 (equivalent to the sample collection included in this meta-analysis) yields close to 100% power to detect the estimated interaction effect of β_GE_ = −0.07 kg/m^2^ per GRS allele from Li *et al* [12]. Under the same assumptions, a sample size of N = 20,000 (roughly equivalent to that of the Li *et al* study [12] yields around 83% power to detect β_GE_ = −0.07 kg/m^2^. Although power to detect the interaction effect from the original study is adequate in the current analysis, we observed a much smaller interaction effect estimate in our meta-analysis (β_GE_ = −0.013 kg/m^2^ per GRS allele), which may be owing to the *Winner's curse* [14]. Indeed, to gain adequate power (80%) to detect this small effect, given the distributions of the GRS and physical activity variables reported in Li *et al*, and assuming that these independent variables are not correlated, would require a sample size considerably larger than the current study (Supplementary Figure S2a).

#### Error, variance and statistical power

We also estimated sample sizes required to detect the interaction between physical activity and the GRS (β_GE_ = −0.07 kg/m^2^ per GRS allele, at 80% power and critical alpha 0.05) when the GRS is dichotomized (GRS </> 11.2 alleles) and all else is held equal; under this scenario, a sample size of approximately 370,000 observations is required (compared with 20,000 observations when the GRS is expressed on a continuum) (Supplementary Figures S2a and S2b), which is owing to the decreased variance in the GRS that occurs with dichotomization (*σ^2^* = 5.06 to *σ^2^* = 0.25) (see Supplementary Table S3 for further details). Loss of power would also be anticipated when a continuous physical activity variable is dichotomized, a concept that is discussed at length elsewhere [15]. We also noted that power to detect the interaction increases as the correlation between the two predictor variables increases, as shown in Supplementary Table S4. The ratio of physically inactive to active persons within a population also influences the variable's variance, and hence sample size requirements; providing the interaction effect is approximately linear, the required sample size is smallest when this ratio is balanced and all else remains equal, as shown in Figure S3.

Combining results from multiple cohorts can also lead to a substantial loss of power owing to inflation of model error. Sources of error may include imprecise measurement of exposures and outcomes [16], variable LD structures between populations, and differences in the magnitude of the relationships of BMI with underlying adiposity phenotypes across populations. In order to account for differences in such error, we compared models based on simulations where the population BMI *σ* increased from 3.5 (as reported in Li *et al*) to, 4.0, 4.5 and 5.5, when all else is held equal. These analyses (Table S5) show that the population *σ* for BMI is inversely related with statistical power to detect the interaction; for example, a sample size of 31,000 yields ∼80% to detect β_GE_ −0.07 kg/m^2^ per GRS allele if the population BMI *σ* = 4.5, whereas the required sample size increases to 46,000 to detect the same effect if the population BMI *σ* = 5.5; a sample size of N∼30,000 is required to achieve 80% power to detect β_GE_ −0.07 kg/m^2^ per GRS allele for the population BMI *σ* = 4.39, as observed in this study.

## Discussion

Here we sought to replicate a widely cited study in which an interaction on BMI was reported between physical activity and a GRS comprised of 12 obesity-predisposing gene variants [12]. The original study is one of the largest and most well conducted single-cohort interaction studies published to date, yet to our knowledge no evidence has been published to show that these findings are replicable. Our study included a collection of cohorts whose sample totaled almost six times the size of the study reported by Li *et al* [12]; the meta-analyzed interaction coefficient is directionally consistent with the original report [12] and statistically significant in the current analysis (*P_interaction_* = 0.015). In secondary analyses, we explored whether any of the individual SNP × physical activity interaction tests were statistically significant; of these, the *FTO* locus (rs1121980) (*P_interaction_* = 0.003), consistent with previous findings [11], and the *SEC16B* rs10913469 variant yielded statistically significant interaction effects (*P_interaction_* = 0.025). The latter finding was not statistically significant after correction for multiple testing, there is no published literature suggesting that this locus is exercise-responsive, and a recent analysis in a randomized clinical trial of lifestyle intervention did not yield evidence of SNP × treatment interactions at the *SEC16B* rs10913469 locus on weight change phenotypes [17], although that analyses was likely underpowered and may be false negative. Thus, validation of the interaction effect observed here for *SEC16B* rs10913469 is necessary to confirm or refute its effect-modifying role for physical activity and obesity.

It is widely acknowledged that initial reports of genetic association signals are often of considerably greater effect magnitude than yielded by subsequent replication attempts; this phenomenon is termed the *Winner's curse* [18]. The large *Winner's curse differential* (Δβ_GE_ = 0.057 kg/m^2^ per GRS allele for the comparison of β_GE_ reported by Li *et al* [12] and observed in the current study) has a dramatic effect on the sample size required for replication, with around 530,000 individuals (>25 times the size of the original study) being required to yield power of 80% to detect the interaction effect reported in this study (β_GE_ = −0.013 kg/m^2^ per GRS allele).

We also conducted a range of simulation analyses to determine how within and between study factors impact power to detect interactions in meta-analyses. We show that the optimal setting is one where i) for a given interaction effect size (β_GE_), the independent variables are expressed on a continuous scale (and if physical activity is dichotomized and the interaction effect is approximately larger the categories should be equally prevalent (i.e., 50%/50%)), ii) the variance in the GRS is large, iii) the GRS and environmental exposure are correlated, and iv) the population variance in the outcome is small, which in part relates to whether exposure and outcome measurements are standardized across studies and measured with reasonable precision (the latter of which is discussed at length elsewhere [16]).

One of the principal arguments for conducting and reporting studies on gene × lifestyle interactions is that they may help identify persons within target populations who are likely to respond well or poorly to specific lifestyle interventions, thus optimizing the delivery and success of the interventions; the same principle may apply to other medical therapies such as drug treatment and surgery. The targeting of lifestyle interventions using genetic information is appealing as it may improve cost-efficiency, reduce harmful side effects, and increase the health-promoting effects of diet and lifestyle factors [19]. However, very few reported gene × lifestyle interactions have been replicated, which may be because many of the original findings were false positive, the reported interaction effects were cohort-specific, or because subsequent studies were underpowered and yielded false negative results [20]. The study by Li *et al* [12] appears well conducted and was performed in a relatively large cohort. The paper was also published in a high impact general medical journal, which implies that the authors' findings are clinically relevant, yet, like most studies of gene × environment interaction, they lacked replication. Importantly, the clinical translation of findings on gene × lifestyle interactions requires that the interaction effect sizes are of a sufficient magnitude to ensure that stratified therapeutic interventions will yield meaningfully different results across genotype groups. The interaction effect size reported in this study is probably too small to be of any clinical value; it is worth noting, though, that in observational studies, where the precision and accuracy with which exposures and outcomes are measured is often low, and where synthetic genetic associations exist (i.e., the observed locus is merely a tag for the latent functional locus), the underlying interaction effect sizes are likely to be underestimated.

A second incentive for conducting studies on gene × lifestyle interactions is that doing so may elucidate biological pathways that lead to the targeting of therapeutic interventions. Most or all of the SNPs studied here probably tag functional variants, with no specific functional role of their own. The functional relevance of the genes most proximal to these SNPs is discussed in detail elsewhere [2]–[6]. The majority of these genes regulate CNS-mediated body weight regulation, energy balance, taste, and satiation [21]; although not clearly established, these genes might also regulate reciprocal behaviors; for example, variants in *MC4R* [22]–[24] and *FTO* [25], [26] are reportedly associated with physical activity.

Although we found statistical evidence of an interaction between physical activity and the GRS in the meta-analysis, it is unlikely that all of the gene variants that comprise the GRS contribute to this interaction effect. For example, the *FTO* variant included in the GRS has been shown previously to interact with physical activity on obesity [11], a finding that was confirmed here, and the *SEC16B* variant also yielded a nominally significant interaction effect in this study. In combination, the two variants yielded an interaction effect size comparable to that seen here for the GRS × physical activity interaction, and the GRS × physical activity interaction test was not statistically significant when the *FTO* and *SEC16B* variants were excluded from the GRS, suggesting that these two loci underlie the aggregate genetic effect of all 12 SNPs combined. It is difficult to accurately speculate on whether the GRS × physical activity interaction reported by Li *et al* [12] is also driven by the *FTO* and *SEC16B* interaction effects, as formal comparisons of this nature were not reported in their paper. Refitting the alleles that comprise a GRS to maximally exploit this information in a regression model (i.e., by weighting the alleles by their interaction effect estimates obtained from SNP × physical activity interaction analyses) would likely increase the magnitude of the observed interaction effect for the GRS; however, to achieve this with minimal bias would require further sample collections to validate these new genetic models, which goes beyond the scope of the present study. Nonetheless, we include the relevant information in Table 2, so that other investigators can construct such weighted models.

It is also important to highlight that the interaction results reported by Li *et al* [12] were not statistically significant once persons with prevalent CVD and cancer were excluded; the inclusion of these individuals may have confounded the interaction effect owing to reporting biases attributable to disease labeling or changes in weight and behavior attributable to the disease processes, although the fact that we have replicated their findings in cohorts that were largely free of these diseases suggests this is not the case. It is also possible that the inclusion of diseased individuals in Li *et al*'s study [12] augmented the interaction effect through hitherto unknown causal mechanisms.

As a general point, it is important to bear in mind that in observational studies, such as those reported here, marginal and interaction effect estimates may not reflect causal processes. This is because physical activity and obesity correlate with other lifestyle, sociodemographic, and metabolic factors, and the gene variants included in the GRS are unlikely to be functional. Thus, even replicated examples of gene × lifestyle interactions may be confounded by latent variables. Reverse causality is a further concern, particularly with cross-sectional data (for example, it is possible that there is a relationship between the GRS and physical activity that is dependent on BMI level).

In summary, our meta-analysis of 111,421 samples from 11 cohorts of European ancestry yielded results that support those of Li *et al* [12]. However, these effects appear evident only when the cohorts from North America (n = 39,810) are included in this meta-analyses. We also demonstrate using simulated data that combining many small cohorts that vary in their classification of physical activity and other factors is a relatively inefficient approach to studying interactions; hence, future studies of gene × lifestyle interactions might prove most effective if focused on a small collection of large cohorts within which standardized and valid lifestyle assessment methods are available.

## Materials and Methods

### Study sample

A total of 111,421 participants from the 11 participating cohorts had genotype and phenotype data necessary for the current analyses. Descriptions of the cohorts included in the current analyses are shown in supplementary Table S6. All participants provided written informed consent and the studies were approved by the relevant institutional review boards and conducted according to the Declaration of Helsinki.

### Body composition and physical activity assessment

In most studies, height and weight were measured using wall-mounted stadiometers and calibrated balance-beam scales, respectively (See Supplementary Table S7). By exception, weight for the NHS, HPFS [27], and WGHS [28] were self-reported. BMI was calculated as weight in kilograms (kg) divided by height in meters squared (m^2^). Obesity was defined according to WHO criteria [29].

Information on physical activity was obtained from self-administered questionnaires, which in most instances were validated. Occupational physical activity in most studies was categorized as i) sedentary or standing; ii) light but partly physically active; iii) light and physically active; and iv) sometimes or often physically straining. Leisure time physical activity during the past three months was categorized as exercising: i) occasionally; ii) 1–2 times/week; iii) 2–3 times/week; or iv) >3 times/week. Among leisure-time physical activity (four categories), participants with missing information were given the lowest intensity score, *i.e.* classified as being ‘occasionally active’. The CPAI was computed by cross-tabulation of occupational and leisure time physical activity, classifying an individual's total physical activity level according to a four-level scale (inactive, moderately inactive, moderately active and active), as previously described [13]. Because some cohorts could not compute the CPAI owing to a lack of specific physical activity data, a binary variable was computed in all cohorts, which classified participants into active (top 80% of the physical activity frequency distribution) and inactive (bottom 20% of the physical activity distribution). This classification most closely matches the frequency distribution obtained when dichotomizing the CPAI variable by combining moderately inactive, moderately active and active individuals (see Supplementary Table S7 for further details), but, as noted in the Results, may not be the most statistically powerful classification.

### Genotyping

DNA was extracted from peripheral blood cells and diluted using standard approaches (see Supplementary Table S8 for further details). Twelve established obesity susceptibility loci [2]–[6] (or their proxies with an *r* ^2^>0.8) were genotyped in the 11 cohorts (Supplementary Table S8). In all cohorts, the genotyping success rates for all 12 variants exceeded 95% and most genotypes were in Hardy-Weinberg equilibrium (*P*>0.001). The exception to this was for the *SH2B1* rs7498665 SNP in the METSIM and HEALTH2006 cohorts, which did not conform to Hardy Weinberg expectations; sensitivity analyses indicated that removing this SNP from the GRS for the METSIM cohort made no material difference to the overall results (data not shown), and so the results shown here are for the full GRS.

### Genetic risk score (GRS)

At each SNP locus, genotypes were coded as 0, 1 and 2 indicating the number of risk alleles (those associated with higher BMI in previous meta-analyses [2]–[6]) and the overall genetic burden for each participant was determined by summing the total number of risk alleles into a GRS, using methods previously described [30].

In cohorts where genotypes were directly assessed (i.e., not imputed from GWAS data), missing genotypes were imputed in participants with four or fewer missing values using previously described methods [31]. Sensitivity analyses performed in the GLACIER and MDC cohorts (n = 39,000) showed that there was no material difference in the effect estimates when analyses were performed with or without imputed genotypes (data not shown), so here only results for the GRS using imputed values are presented. The GRS was normally distributed in all cohorts.

### Statistical analysis

Statistical analyses were performed using the SAS software (SAS Institute, Cary, NC), *R* software (http://www.r-project.org/) and STATA (version 12, StataCorp, College Station, TX, USA). General linear models (GLM) were used to test the association of the GRS with BMI. Logistic regression was used to test genetic associations with obesity. All analyses were adjusted for age, age^2^, sex, study center (for multi-center studies), and physical activity (where appropriate), and we assumed additive effects of the alleles. Interaction tests for individual SNPs and the GRS with physical activity (for outcomes BMI or obesity) were performed by including a SNP (or GRS) × physical activity interaction term in the model, with the marginal effect terms also included. The genetic effect estimates for BMI were also calculated by strata of physical activity (i.e. inactive vs. combined active), as described above.

### Meta-analysis

Meta-analyses were undertaken using the *metan* command in STATA (version 12, StataCorp, College Station, TX, USA). A summary interaction effect estimate was calculated for all 11 cohorts combined using meta-analysis weighted by cohort sample size to summarize the pairwise (SNP/GRS × physical activity) interaction coefficients and SE derived from each cohort. Meta-analyses were repeated using random and fixed effects models, but between-study heterogeneity was low (χ^2^ = 15.51, *I^2^* = 3.3% and *P*-val = 0.415); thus, the results were not materially different to the weighted approach (data not shown), leading us to present only the weighted results here. Analysis of data from the InterAct Study, which includes multiple sub-cohorts, was conducted as described elsewhere [32]. The full InterAct Study includes two Swedish study centers in Malmö and Umeå, which overlap extensively with the GLACIER and MDC cohorts. Thus, these Swedish InterAct cohort samples were not included in the main analyses.

### Statistical power

The code-generating program mlPowSim [33] was used to generate *R* code for simulations and power estimation with 1,000 iterations for each sample size simulation. In order to estimate power for different samples sizes, we simulated a 12 SNP GRS using a random normal distribution with mean (s.d.) 11.2 (2.2); physical activity was simulated using a binomial distribution assuming the population prevalence of physical inactivity was 30%, as estimated by Li *et al.* The approach (described in detail in the Supplementary Material S1) was used to simulate different scenarios for the predictor variables: i) with the GRS expressed as a continuous or dichotomized variable (Supplementary Figures S2a and S2b), ii) a range of frequencies for the binary physical activity variable and variances (*σ^2^*) (Figure S3, iii) a range of effect sizes for β_GE_ (Supplementary Figures S2a and S2b), iv) a range of covariances between the two predictor variables (Figure S3), and v) a range of variances (*σ^2^*) for the population (Supplementary Table S5).

The main power calculations were performed using estimates obtained from Li *et al* [12]: a GRS marginal effect (β_G_) of 0.154 kg/m^2^ per GRS risk allele and a physical activity marginal effect (β_E_) of −0.313 kg/m^2^ (active vs. inactive), physical inactivity prevalence of 30%, and s.d of ±3.5. We assumed that the GRS and physical activity are not correlated and a two-sided critical alpha of 0.05 was used in the calculations. Although the interaction effect estimate (β_GE_) is not explicitly reported in Li *et al's* paper, we were able to estimate this from the GRS effect estimates reported in Table 2 of their paper (β_GE_∼−0.07) by approximating the difference of β_G_ between the two combined activity categories (active vs. inactive). To accommodate imprecision in the estimation of β_GE_ and the possibility that Li *et al's* study [12] was affected by the ‘winner's curse’ [18] and thus over-estimated the interaction effect size one could hope to observe in other cohorts, we show statistical power estimations for interaction effects ranging from −0.05 to −0.10 (Supplementary Figure S2a). We also simulated the GRS as a binary variable and compared power using this approach with one where the GRS is expressed on a continuum (Supplementary Figure S2b), as GRSs are often reported on the binary scale in genetic association studies.

## Supporting Information

Figure S1 Forest plot showing the meta-analysis of interaction coefficients (GRS × Cambridge Physical Activity Index) in relation to BMI in the three North American cohorts (a) and the meta-analysis of interaction coefficients (GRS × Cambridge Physical Activity Index) in relation to BMI in the eight European cohorts (b).
(TIF)

Figure S2 Sample size and power to detect an interaction (β_GE_ = −0.013 to −0.10) between a normally distributed genetic risk score (expressed on a continuous [panel A] or binary [panel B] scale) and physical activity (30% inactive and 70% active). Critical alpha = 0.05. All other parameters are taken from Li et al [4].
(TIF)

Figure S3 Sample size required for 80% power to detect a gene × physical activity interaction in obesity when the prevalence of physical activity (and the variable's variance) varies and all other parameters are fixed. Mean and variance of the genetic risk score are set at 11.2 and 5.06 respectively. Statistical power and critical alpha are fixed at 80% and 0.05 respectively. Solid line represents required sample sizes, dashed line represents *σ^2^* for corresponding prevalence of physical activity, and dotted lines mark the 50^th^ and 80^th^ centile cut-points and the respective sample size requirements for the binary physical activity variable. Power calculations assume a linear interaction effect.
(TIF)

Material S1 Additional details on statistical power simulation.
(DOC)

Table S1 Cohort-specific descriptive statistics.
(DOC)

Table S2 Interactions between the 12 SNPs and CPAI (4 level scale) on BMI across each of the 11 cohorts.
(DOC)

Table S3 Power to detect gene × physical activity interaction in obesity for the different simulation settings: physical activity is a binary variable, and variance of genetic risk score varies.
(DOC)

Table S4 Power to detect a gene × physical activity interaction in obesity for the different simulations settings: physical activity is either binary or approximated by a normal distribution and with different degrees of correlation between the physical activity variable and the genetic risk score.
(DOC)

Table S5 Sample sizes required to detect an interaction between a genetic risk score (12 SNPs) and physical activity (binary) when the standard deviation (S.D.) in the outcome (BMI) varies and all other parameters are fixed.
(DOC)

Table S6 Study description of participating cohorts.
(DOC)

Table S7 Cohort-specific methods used for measuring body mass index and physical activity.
(DOC)

Table S8 Genotyping methods and SNP quality control.
(DOC)
