# Supplementary figures and images for: Gene × Physical Activity Interactions in Obesity: Combined Analysis of 111,421 Individuals of European Ancestry
Source: PLoS Genet. 2013 Jul 25;9(7):e1003607. doi: 10.1371/journal.pgen.1003607 (PMC3723486; doi:10.1371/journal.pgen.1003607)

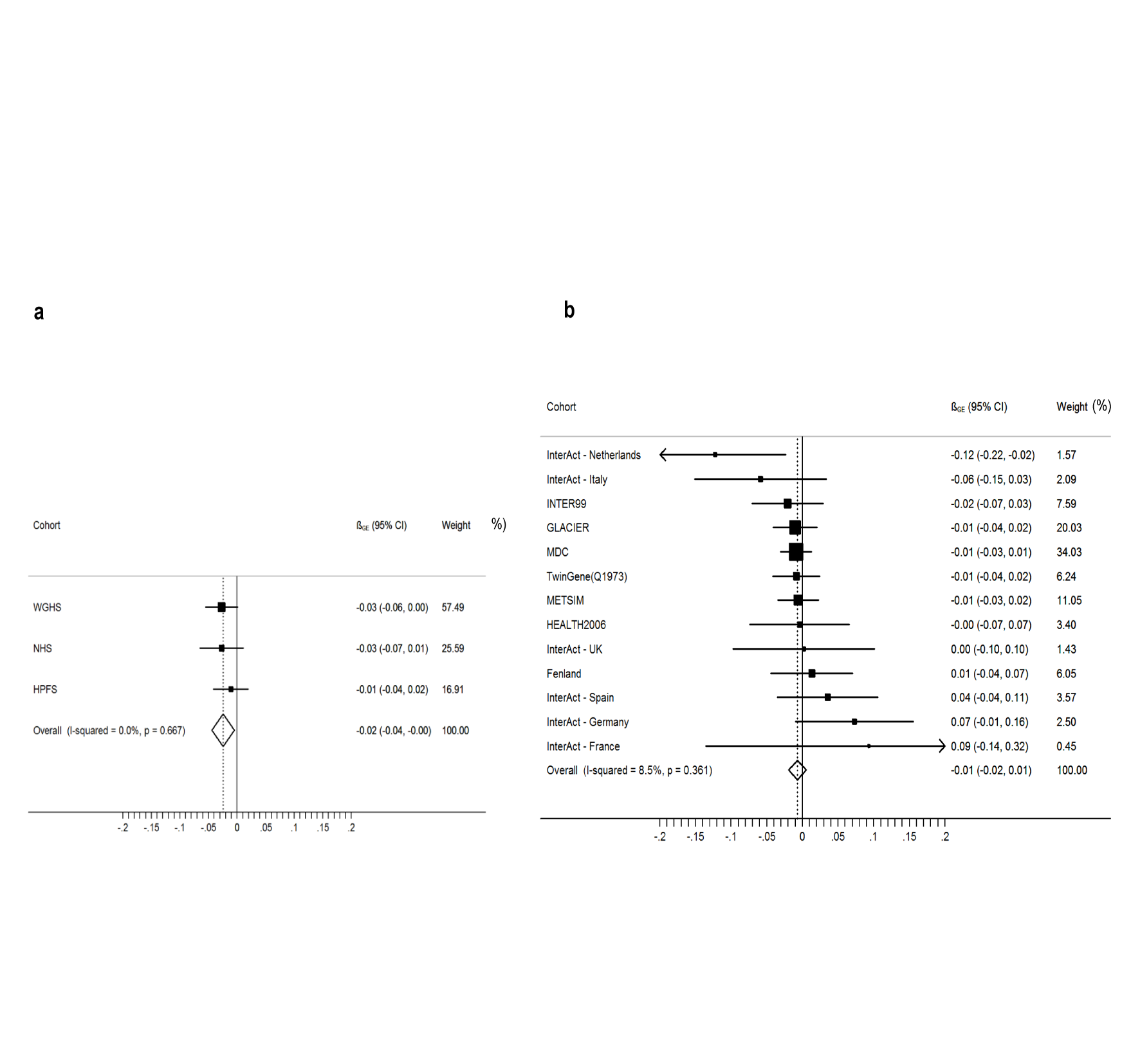

Supplement: Figure S1 — Forest plot showing the meta-analysis of interaction coefficients (GRS × Cambridge Physical Activity Index) in relation to BMI in the three North American cohorts (a) and the meta-analysis of interaction coefficients (GRS × Cambridge Physical Activity Index) in relation to BMI in the eight European cohorts (b). (TIF) [file pgen.1003607.s001.tif]

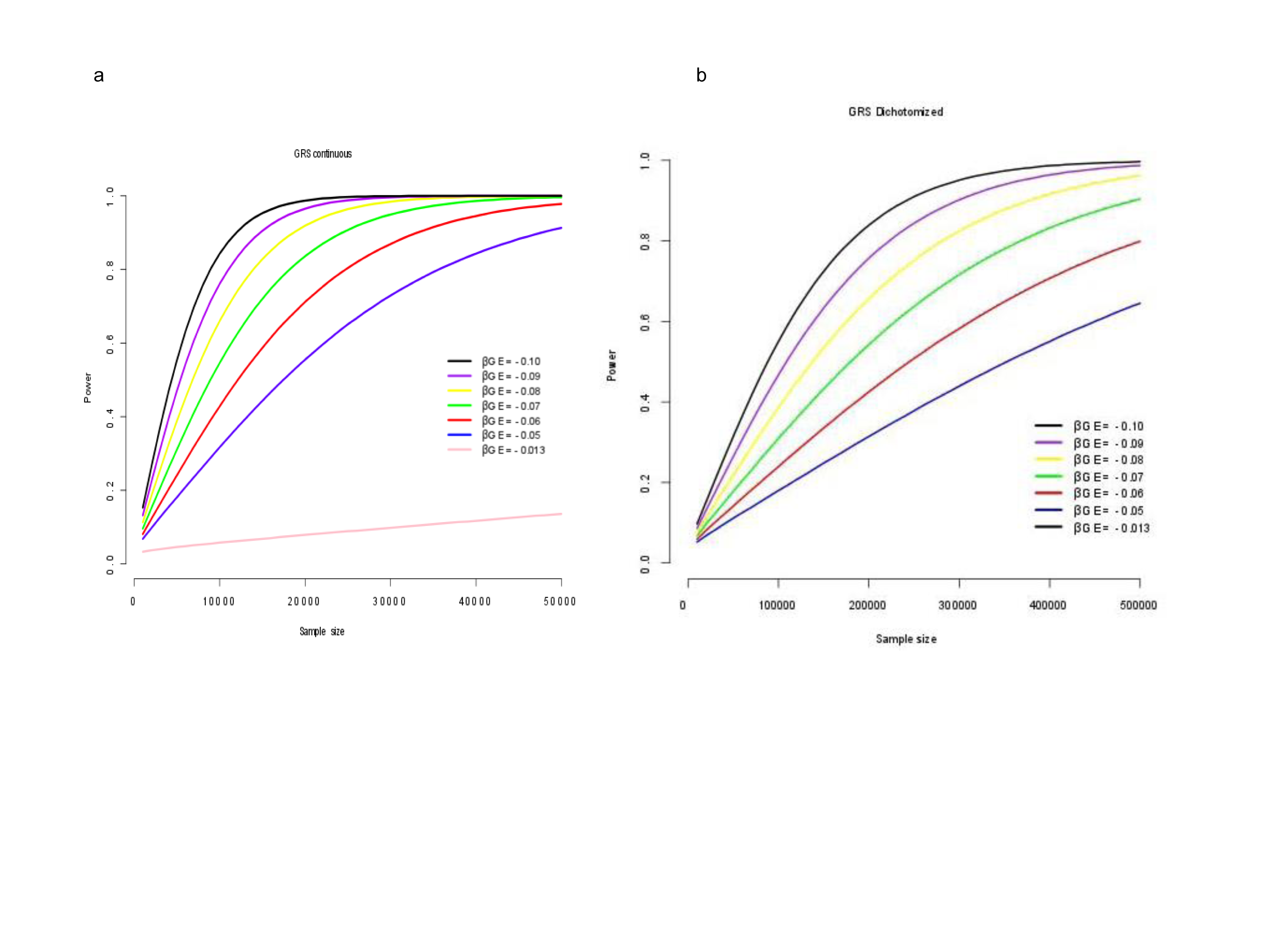

Supplement: Figure S2 — Sample size and power to detect an interaction (βGE = −0.013 to −0.10) between a normally distributed genetic risk score (expressed on a continuous [panel A] or binary [panel B] scale) and physical activity (30% inactive and 70% active). Critical alpha = 0.05. All other parameters are taken from Li et al [4]. (TIF) [file pgen.1003607.s002.tif]

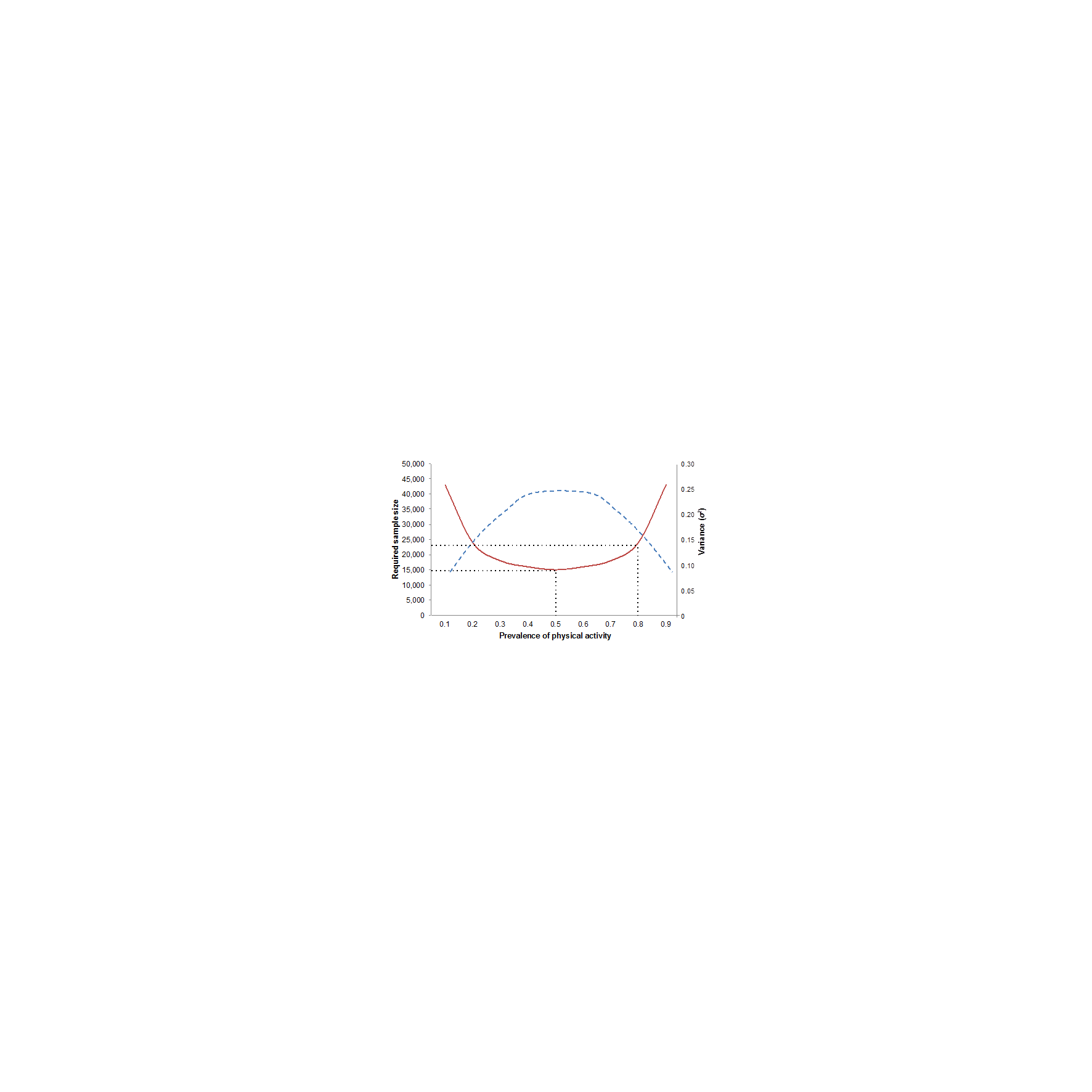

Supplement: Figure S3 — Sample size required for 80% power to detect a gene × physical activity interaction in obesity when the prevalence of physical activity (and the variable's variance) varies and all other parameters are fixed. Mean and variance of the genetic risk score are set at 11.2 and 5.06 respectively. Statistical power and critical alpha are fixed at 80% and 0.05 respectively. Solid line represents required sample sizes, dashed line represents σ2 for corresponding prevalence of physical activity, and dotted lines mark the 50th and 80th centile cut-points and the respective sample size requirements for the binary physical activity variable. Power calculations assume a linear interaction effect. (TIF) [file pgen.1003607.s003.tif]
