# Supplementary material for: Gene × Physical Activity Interactions in Obesity: Combined Analysis of 111,421 Individuals of European Ancestry
Source: PLoS Genet. 2013 Jul 25;9(7):e1003607. doi: 10.1371/journal.pgen.1003607 (PMC3723486; doi:10.1371/journal.pgen.1003607)
Supplement: Material S1 — Additional details on statistical power simulation. (DOC) [file pgen.1003607.s004.doc]

**Material S1.** Additional details on statistical power simulation.

**Power calculation**

In this appendix we present details of simulation-based power calculations. We focus on a simple interaction regression model with fixed effects, where is the value of the response variable and are the known constants of the independent variables and are random errors for is population variance and ’s are regression parameters. For the purpose of detecting the interaction effect the problem is concerned with its least squares estimator and distributional property of the corresponding test statistics for testing the hypothesis against Details about the parameter estimation and testing the hypotheses in multiple linear regression are given elsewhere [1,2]. We computed power using the equation where is the effect size, is the standard error of, and are the significance threshold and the power of the test, respectively. These four quantities are linked by the equation under the assumption of a normal distribution for the effect size, and yield approximate estimates under other non-normal distributions. Further, depends on the sample size, the population variance and the distributions of the independent variables. Given the ‘true’ effect size, we use the average of the estimated standard errors over the set of simulations and to solve the equation and calculate statistical power. Modified *R* code produced by MLPowSim [3] and the reported values of the effect sizes and population variance in [4] as well as the distribution parameters of the independent variables, are used to execute calculations.

Briefly, values for the GRS and physical activity variables were randomly drawn from the relevant distributions. The GRS variable (*G* above) was centered to zero and multiplied by the physical activity variable to obtain a GRS × physical activity interaction variable. The outcome variable (BMI in this instance) was calculated according to the regression formula above. The intercept was estimated from Li *et al* Figure 3 [4], and random error simulated from a random normal distribution with mean (s.d.) of 0(σ).

**Some simulation scenarios**

We performed simulations for several relevant scenarios with the input parameters reported in Li et al: and. Genetic risk score (G) is assumed to be normally distributed and physical activity (E) is either binary when it is used as the categorical independent variable/predictor in the model or can be approximated by a normal distribution when the correlation between predictors are simulated. In this scenario, the joint bivariate normal distribution is given by were , and being correlation and covariance between the and respectively.

A positive correlation between the predictor variables increases power to detect the interaction (i.e., the greater the correlation between the independent variables the higher the statistical power to detect interaction) (Supplementary Table S4). For the fixed sample size, statistical power decreases concomitantly with a declining variance of the predictor variable (Supplementary Tables S3). The sample size required for the fixed power (of 0.8) increases as the variance of the predictor variable decreases (Supplementary Table S3, Figure S3) and the population variance increases (Supplementary Table S5).

**Online Supplementary Information references**

1. Jaccard JJ, Robert T (2005) Interaction effects in Multiple regression. Sage Publications (CA).

2. Montgomery DC (2008) Design and Analysis of Experiments. John Wiley & Sons.

3. Browne WJ, Golalizadeh LM, Parker RMA (2009) Sample Size, Identifiability and MCMC Efficiency in Complex Random Effect Models. Bristol 150 p.

4. Li S, Zhao JH, Luan J, Ekelund U, Luben RN, et al. (2010) Physical activity attenuates the genetic predisposition to obesity in 20,000 men and women from EPIC-Norfolk prospective population study. PLoS Med 7.pii: e1000332.
