# Supplementary material for: Gene × Physical Activity Interactions in Obesity: Combined Analysis of 111,421 Individuals of European Ancestry
Source: PLoS Genet. 2013 Jul 25;9(7):e1003607. doi: 10.1371/journal.pgen.1003607 (PMC3723486; doi:10.1371/journal.pgen.1003607)
Supplement: Table S1 — Cohort-specific descriptive statistics. (DOC) [file pgen.1003607.s005.doc]

**Table S1.** Cohort-specific descriptive statistics

| **Study** | **N** | **Sex**  (% Female) | **Age**  (years)* | **BMI**  (kg/m²)* | **GRS**  (alleles)* |
| --- | --- | --- | --- | --- | --- |
| FENLAND | 4,330 | 54 | 46.3 (7.2) | 26.8 (4.8) | 11.4 (2.2) |
| GLACIER | 14,341 | 61 | 51.8 (8.8) | 25.8 (4.0) | 12.3 (2.2) |
| HEALTH 2006 | 2,437 | 55 | 49.2 (12.9) | 25.8 (4.6) | 11.4 (2.3) |
| HPFS | 6,733 | 0 | 55.1 (8.7) | 25.8 (3.3) | 11.3 (2.2) |
| INTER99 | 5,438 | 51 | 46.0 (7.9) | 26.1 (4.4) | 11.4 (2.2) |
| INTERACT | 8,317 | 66 | 51.2 (9.2) | 26.3 (4.3) | 11.1 (2.2) |
| MDC | 24,368 | 60 | 58.1 (7.7) | 25.7 (4.0) | 12.3 (2.2) |
| METSIM | 7,911 | 0 | 57.1 (7.1) | 26.8 (3.8) | 10.7 (2.1) |
| NHS | 10,189 | 100 | 54.1 (6.7) | 25.8 (5.1) | 11.3 (2.2) |
| TWINGENE (Q2000) | 8,025 | 54 | 64.1 (7.2) | 25.9 (3.9) | 11.5 (2.2) |
| TWINGENE (Q1973) | 4,469 | 55 | 30.3 (7.8) | 21.8 (2.6) | 11.5 (2.2) |
| WGHS | 22,888 | 100 | 54.7 (7.1) | 25.9 (5.0) | 11.3 (2.2) |

*Age, BMI and GRS given as mean (SD). Although TWINGENE is a single study, baseline data on physical activity are taken from two distinct time points; thus the study is shown here for these sub-cohorts separately
