# Supplementary material for: Gene × Physical Activity Interactions in Obesity: Combined Analysis of 111,421 Individuals of European Ancestry
Source: PLoS Genet. 2013 Jul 25;9(7):e1003607. doi: 10.1371/journal.pgen.1003607 (PMC3723486; doi:10.1371/journal.pgen.1003607)
Supplement: Table S2 — Interactions between the 12 SNPs and CPAI (4 level scale) on BMI across each of the 11 cohorts. (DOC) [file pgen.1003607.s006.doc]

**Table S2. Interactions between the 12 SNPs and CPAI (4 level scale) on BMI across each of the 11 cohorts**

| **Lead SNP** | **Nearest gene** | **GLACIER** | **MDC** | **NHS** | **HPFS** | **INTER99** | **HEALTH 2006** | **TWINGENE (Q1973)** | **METSIM** | **INTERACT** | **FENLAND** | **WGHS** |
| --- | --- | --- | --- | --- | --- | --- | --- | --- | --- | --- | --- | --- |
| rs1121980 | *FTO* | *0.09 (0.05); P=0.071 | *-0.04 (0.04); P=0.211 | -0.11 (0.06); P=0.079 | -0.05 (0.05); P=0.335 | -0.17 (0.08); P=0.041 | -0.12 (0.12); P=0.313 | 0.04 (0.07); P=0.558 | 0.02 (0.04); P=0.618 | -0.011 (0.05); P=0.8291 | *0.15 (0.09); P=0.114 | -0.16 (0.05); P=0.001 |
| rs7498665 | *SH2B1* | -0.03 (0.05); P=0.585 | 0.01 (0.04); P=0.844 | -0.04 (0.06); P=0.507 | -0.03 (0.05); P=0.608 | 0.03 (0.08); P=0.702 | 0.1 (0.12); P=0.417 | -0.01 (0.05); P=0.803 | 0.16 (0.09); P=0.086 | 0.13 (0.05); P=0.012 | *-0.05 (0.09); P=0.61 | -0.07 (0.05); P=0.15 |
| rs10913469 | *SEC16B* | -0.07 (0.06); P=0.236 | -0.03 (0.04); P=0.526 | -0.07 (0.08); P=0.34 | -0.13 (0.06); P=0.043 | *0.05 (0.1); P=0.608 | *0.01 (0.13); P=0.919 | 0.01 (0.07); P=0.891 | -0.07 (0.06); P=0.222 | *0.02 (0.07); P=0.802 | *-0.05 (0.11); P=0.638 | -0.06 (0.06); P=0.298 |
| rs10838738 | *MTCH2* | *-0.11 (0.05); P=0.033 | -0.04 (0.04); P=0.231 | 0.08 (0.06); P=0.23 | 0.03 (0.05); P=0.532 | 0.11 (0.09); P=0.214 | 0.12 (0.12); P=0.31 | -0.06 (0.06); P=0.264 | -0.03 (0.05); P=0.5 | -0.003 (0.05); P=0.954 | *0.06 (0.09); P=0.529 | -0.02 (0.05); P=0.654 |
| rs17782313 | *MC4R* | -0.03 (0.06); P=0.53 | -0.03 (0.04); P=0.4 | 0 (0.07); P=0.989 | -0.05 (0.06); P=0.354 | -0.16 (0.09); P=0.086 | -0.01 (0.13); P=0.918 | 0.02 (0.05); P=0.75 | -0.05 (0.06); P=0.346 | 0.14 (0.06); P=0.018 | *-0.07 (0.11); P=0.514 | -0.01 (0.05); P=0.799 |
| rs3101336 | *NEGR1* | *0.01 (0.05); P=0.875 | *0.01 (0.04); P=0.802 | -0.08 (0.06); P=0.224 | -0.03 (0.05); P=0.54 | 0.05 (0.08); P=0.526 | 0.08 (0.12); P=0.482 | 0.03 (0.06); P=0.606 | *0.07 (0.05); P=0.133 | -0.005 (0.05); P=0.9242 | *0.02 (0.1); P=0.814 | 0 (0.05); P=0.965 |
| rs6548238 | *TMEM18* | 0.1 (0.06); P=0.103 | 0.03 (0.05); P=0.565 | -0.02 (0.08); P=0.814 | -0.04 (0.06); P=0.541 | -0.2 (0.11); P=0.057 | -0.02 (0.15); P=0.918 | -0.05 (0.05); P=0.379 | 0.09 (0.06); P=0.128 | -0.22 (0.07); P=0.0016 | *0.03 (0.12); P=0.796 | -0.01 (0.06); P=0.833 |
| rs10938397 | *GNPDA2* | -0.02 (0.05); P=0.691 | -0.02 (0.04); P=0.547 | 0.02 (0.06); P=0.804 | -0.01 (0.05); P=0.79 | 0.1 (0.08); P=0.211 | -0.01 (0.12); P=0.953 | -0.06 (0.06); P=0.309 | -0.03 (0.04); P=0.518 | 0.049 (0.05); P=0.356 | -0.03 (0.09); P=0.778 | 0 (0.05); P=0.972 |
| rs925946 | *BDNF* | *-0.1 (0.06); P=0.086 | *0 (0.04); P=0.983 | -0.05 (0.07); P=0.45 | 0 (0.05); P=0.999 | -0.08 (0.09); P=0.352 | -0.13 (0.12); P=0.313 | -0.02 (0.05); P=0.724 | -0.01 (0.05); P=0.869 | 0.015 (0.06); P=0.792 | -0.03 (0.1); P=0.779 | 0.05 (0.05); P=0.258 |
| rs368794 | *KCTD15* | *0 (0.05); P=0.933 | *0.02 (0.04); P=0.501 | -0.01 (0.06); P=0.87 | 0.02 (0.05); P=0.68 | -0.02 (0.09); P=0.824 | -0.07 (0.12); P=0.566 | -0.04 (0.06); P=0.484 | *-0.06 (0.05); P=0.224 | 0.04 (0.05); P=0.514 | *-0.01 (0.1); P=0.937 | 0 (0.05); P=0.999 |
| rs7647305 | *ETV5* | 0.02 (0.06); P=0.731 | 0.08 (0.04); P=0.095 | 0.05 (0.08); P=0.539 | 0.05 (0.06); P=0.393 | 0.06 (0.1); P=0.526 | -0.08 (0.14); P=0.556 | 0.01 (0.05); P=0.908 | -0.01 (0.06); P=0.791 | 0.02 (0.06); P=0.812 | *0.02 (0.11); P=0.879 | -0.04 (0.05); P=0.502 |
| rs7132908 | *FAIM2* | *0.03 (0.05); P=0.585 | *-0.06 (0.04); P=0.111 | -0.07 (0.06); P=0.26 | 0.07 (0.05); P=0.157 | *-0.12 (0.08); P=0.158 | *0.04 (0.12); P=0.736 | 0.04 (0.07); P=0.593 | 0 (0.04); P=0.999 | *-0.03 (0.05); P=0.513 | *0.01 (0.1); P=0.883 | -0.01 (0.05); P=0.85 |

Interactions shown as Beta (SE); P-Value. * indicates a proxy SNP was used, further details on the proxies used within each study can be found in Table S8
