# Supplementary material for: Gene × Physical Activity Interactions in Obesity: Combined Analysis of 111,421 Individuals of European Ancestry
Source: PLoS Genet. 2013 Jul 25;9(7):e1003607. doi: 10.1371/journal.pgen.1003607 (PMC3723486; doi:10.1371/journal.pgen.1003607)
Supplement: Table S3 — Power to detect gene × physical activity interaction in obesity for the different simulation settings: physical activity is a binary variable, and variance of genetic risk score varies. (DOC) [file pgen.1003607.s007.doc]

**Table S3. Power to detect a gene x physical activity interaction in obesity for the different simulation settings: physical activity is a binary variable, and variance of the genetic risk score varies**

|  |  |
| --- | --- |
| 5.06 | 0.837 |
| 2.5 | 0.542 |
| 1 | 0.257 |
| 0.5 | 0.15 |

Where is Power, is variance of the genetic risk score. Prevalence of physical activity is set at 0.7. Sample size is fixed at 20,000 and the BMI (population) S.D. is set to 3.5
