# Supplementary material for: Gene × Physical Activity Interactions in Obesity: Combined Analysis of 111,421 Individuals of European Ancestry
Source: PLoS Genet. 2013 Jul 25;9(7):e1003607. doi: 10.1371/journal.pgen.1003607 (PMC3723486; doi:10.1371/journal.pgen.1003607)
Supplement: Table S4 — Power to detect a gene × physical activity interaction in obesity for the different simulations settings: physical activity is either binary or approximated by a normal distribution and with different degrees of correlation between the physical activity variable and the genetic risk score. (DOC) [file pgen.1003607.s008.doc]

**Table S4.** Power to detect a gene x physical activity interaction in obesity for the different simulations settings: physical activity is either a binary variable or approximated by a normal distribution and with different degrees of correlation between the physical activity variable and the genetic risk score

| E distribution |  |  |  |
| --- | --- | --- | --- |
| E binary | 0 | 0 | 0.837 |
| E normal | 0 | 0 | 0.837 |
| E normal | 0.1 | 0.12 | 0.842 |
| E normal | 0.5 | 0.62 | 0.929 |

Where E is physical activity, with prevalence set at 0.7and are the correlation and covariance between physical activity and the genetic risk score respectively. is power. Sample size is fixed at 20,000 and the BMI (population) S.D. is set to 3.5. Mean and variance of the genetic risk score are set at 11.2 and 5.06 respectively.
