# Supplementary material for: Gene × Physical Activity Interactions in Obesity: Combined Analysis of 111,421 Individuals of European Ancestry
Source: PLoS Genet. 2013 Jul 25;9(7):e1003607. doi: 10.1371/journal.pgen.1003607 (PMC3723486; doi:10.1371/journal.pgen.1003607)
Supplement: Table S5 — Sample sizes required to detect an interaction between a genetic risk score (12 SNPs) and physical activity (binary) when the standard deviation (S.D.) in the outcome (BMI) varies and all other parameters are fixed. (DOC) [file pgen.1003607.s009.doc]

**Table S5.** Sample sizes required to detect an interaction between a genetic risk score (12 SNPs) and physical activity (binary) when the standard deviation (S.D.) in the outcome (BMI) varies and all other parameters are fixed

| S.D. | Sample size for 80% power |
| --- | --- |
| 3.50 | 19,000* |
| 4.00 | 25,000 |
| 4.39 | 30,000** |
| 4.50 | 31,000 |
| 5.00 | 38,000 |
| 5.50 | 46,000 |

* From Li *et al*, PLoS Medicine, 2010 (N=20,00)

** From replication study of Li *et al* (N=110,000)
