# Supplementary material for: Gene × Physical Activity Interactions in Obesity: Combined Analysis of 111,421 Individuals of European Ancestry
Source: PLoS Genet. 2013 Jul 25;9(7):e1003607. doi: 10.1371/journal.pgen.1003607 (PMC3723486; doi:10.1371/journal.pgen.1003607)
Supplement: Table S6 — Study description of participating cohorts. (DOC) [file pgen.1003607.s010.doc]

**Table S6.** Study description of participating cohorts.

| **Cohort** | **Study description** | **Relevant Reference**  **(PubMed ID)** |
| --- | --- | --- |
| FENLAND (UK) | The Fenland Study is an ongoing, population-based cohort study (started in 2005) designed to investigate the association between genetic and lifestyle environmental factors and the risk of obesity, insulin sensitivity, hyperglycemia and related metabolic traits in men and women born in the years 1950 to 1975. Potential volunteers were recruited from General Practice sampling frames in the Fenland, Ely and Cambridge areas of the Cambridgeshire Primary Care Trust in the UK. Exclusion criteria for the study were: prevalent diabetes, pregnant and lactating women, inability to participate due to terminal illness, psychotic illness, or inability to walk unaided. All participants had measurements done at the MRC Epidemiology Unit Clinical Research Facilities in Ely, Wisbech and Cambridge. Participants attended after an overnight fast for a detailed clinical examination, and blood samples were collected. The Local Research Ethics Committee granted ethical approval for the study and all participants gave written informed consent. | (22708638) |
| GLACIER  (Sweden) | The Gene-Lifestyle interactions And Complex traits Involved in Elevated disease Risk (GLACIER) Study is nested within the Västerbotten Health Survey, which is part of the Northern Sweden Health and Disease Study, a population-based prospective cohort study from northern Sweden. A total of 14,341 participants from the GLACIER Study had complete genotype and phenotype data necessary for the current analyses. | (20870969)  (14660243) |
| HEALTH 2006  (Denmark) | Health2006 is a population-based epidemiological study of general health, diabetes and cardiovascular disease of 3,471 individuals aged 18-74 years conducted at the Research Centre for Prevention and Health in Glostrup, Denmark. | (22374641) |
| HPFS  (USA) | The Health Professionals Follow-up Study (HPFS) is a prospective cohort study of 51,529 U.S. male health professionals who were 40 to 75 years old at study inception in 1986. Information about medical history, lifestyle and health conditions has been collected biennially by self-administered questionnaires every 2 years since inception. | (1678444) |
| INTER99  (Denmark) | The Inter99 cohort is a randomized, non-pharmacological intervention study for the prevention of ischemic heart disease, conducted on 6,784 randomly ascertained participants aged 30 to 60 years at the Research Centre for Prevention and Health in Glostrup, Denmark | (23457408) |
| INTERACT | InterAct is a large, well-powered, prospective study that aims to improve understanding of the interplay between genes and lifestyle factors on the risk of type 2 diabetes development. A total of 12,403 verified incident cases of type 2 diabetes occurred during 3.99 million person-years of follow-up of 340,234 EPIC participants eligible for InterAct. InterAct cases were followed-up for an average of 6.9 years. | (21717116) |
| MDC  (Sweden) | The population-based Malmö Diet and Cancer (MDC) cohort with baseline examinations from March 1991 to October 1996 consist of 30,447 individuals. All men born 1923-1945 and all women born 1923-1950 and living in Malmö were invited to participate. The total number of MDC participants eligible for the current analyses was 24,368. | (8429286) |
| METSIM  (Finland) | The Metabolic Syndrome in Men (METSIM) study is a population-based cross-sectional study comprising a total of 10,197 men. Participants, aged from 45 to 70 years, were randomly selected from the population register of Kuopio, Eastern Finland (population of 95,000) and examined within years 2005-2010. | (22486802) |
| NHS  (USA) | The Nurses’ Health Study (NHS) is a prospective cohort study of 121,700 female registered nurses who were 30 to 55 years old at study inception in 1976. Information about medical history, lifestyle and health conditions has been collected biennially by self-administered questionnaires every 2 years since inception. | (9065374) |
| TWINGENE (Q1973) & (Q2000)  (Sweden) | The TwinGene project, conducted between 2004 and 2008, is a population-based Swedish study of twins born between 1911 and 1958. The study participants have previously participated in a telephone interview called Screening Across the Lifespan Twin Study, conducted between 1998 and 2002. To be included in TwinGene, both twins within a pair had to be alive. The zygosity of the twins was based on self-reported childhood resemblance, or by using DNA markers (for 18% of the total sample). In total, 12,591 individuals participated by donating blood to the study, and by answering questionnaires about life style and health. | (23137839) |
| WGHS  (USA) | The Women’s Genome Health Study (WGHS) is a prospective cohort of initially healthy, female North American health care professionals at least 45 years old at baseline representing participants in the Women’s Health Study (WHS) who provided a blood sample at baseline and consent for blood-based analyses. The WHS was a 2x2 trial beginning in 1992-1994 of vitamin E and low dose aspirin in prevention of cancer and cardiovascular disease with about 10 years of follow-up. Since the end of the trial, follow-up has continued in observational mode. Additional information related to health and lifestyle were collected by questionnaire throughout the WHS trial and continuing observational follow-up. | (18070814) |
