# Supplementary material for: Gene × Physical Activity Interactions in Obesity: Combined Analysis of 111,421 Individuals of European Ancestry
Source: PLoS Genet. 2013 Jul 25;9(7):e1003607. doi: 10.1371/journal.pgen.1003607 (PMC3723486; doi:10.1371/journal.pgen.1003607)
Supplement: Table S7 — Cohort-specific methods used for measuring body mass index and physical activity. (DOC) [file pgen.1003607.s011.doc]

**Table S7.** Cohort-specific methods used for measuring body mass index and physical activity.

| **Cohort** | **Physical activity** | **Cambridge physical activity index (CPAI)** | **Binary physical activity** | **Body mass index (kg/m^2^)** |
| --- | --- | --- | --- | --- |
| FENLAND | Physical activity was assessed using the Recent Physical Activity Questionnaire (RPAQ, Besson et al. Am J Clin Nutr 2010 Jan;91(1):106-14). This is aimed at assessing physical activity in the past 4 weeks based on self-reported activity in four main domains: at home, at work, during transport and during leisure time. Total activity volume were estimated by multiplying frequency and duration (in hours per day) with the net metabolic cost of each activity, expressed in metabolic equivalents (METs), obtained from the PA compendium (Ainsworth et al Med Sci Sports Exerc 2000;32:S498–504; Ainsworth et al. Med Sci Sports Exerc 1993;25:71–80 | Physical activity was categorised into four levels using sex-specific quartiles of physical activity volume | Physical activity was categorized into two levels comparing the "most inactive" individuals (classified as those in the lowest quintile of physical activity) with all others. | Weight was measured to the nearest 200 g with a calibrated electronic scale (TANITA model BC-418 MA; Tanita, Tokyo, Japan). Height was assessed to the nearest 0.1 cm with a wall-mounted stadiometer (SECA 240; Seca, Birmingham, United Kingdom). Body mass index (BMI; in kg/m2) was calculated as weight divided by height squared. |
| GLACIER | Occupational physical activity categorized as i) sedentary or standing; ii) light but partly physically active; iii) light and physically active; iv) sometimes physically straining; or v) physically straining most of the time) and leisure time physical activity during the past three months (categorized as exercising: i) never; ii) occasionally; iii) 1-2 times/week; iv) 2-3 times/week; or v) >3 times/week) were assessed with a validated self-administered questionnaire. Among occupational physical activity (five categories), ii and iii were collapsed into a single category and similarly for leisure time physical activity (five categories), i and ii were collapsed. The mean intensity score was used in case of multiple answers. | The CPAI was computed by cross-tabulation of occupational and leisure time physical activity, classifying an individual’s total physical activity level according to a four-level scale (inactive, moderately inactive, moderately active and active). Participants with missing information for either leisure time or occupational physical activity were given the lowest intensity score before cross-tabulation. In sensitivity analyses, excluding these individuals from the analyses didn’t materially alter the results. | Participants with a CPAI=1 were classified as inactive and the remaining participants were classified as active (CPAI 2-4) | Height and weight were measured using wall-mounted stadiometers and calibrated balance-beam scales, respectively. BMI was calculated as weight in kilograms (kg) divided by height in meters squared (m^2^) |
| HEALTH 2006 | The level of physical activity was assessed using questionnaire data. Occupational physical activity was categorized as i) sedentary or standing ii) light but partly physically active iii) physically active iv) physically straining most of the time v) not working. Leisure-time physical activity was categorized as i) sedentary or minimal physical activity ii) light physical activity iii) modest physical activity iv) highly vigorous physical activity. Among occupational physical activity (five categories) i and v were collapsed into a single category. | The CPAI was computed by cross-tabulation of occupational and leisure-time physical activity, classifying an individual’s total physical activity level according to a four-level scale (inactive, moderately inactive, moderately active and active). Among leisure-time physical activity, participants with missing information were given the lowest intensity score, i.e. classified as being ‘occasionally active’. | Participants with a CPAI=1 were classified as inactive and the remaining participants were classified as active (CPAI 2-4) | Height (cm) and body weight (kg) was measured in light indoor clothes and without shoes. BMI was calculated as weight in kilograms (kg) divided by height in meters squared (m^2^). |
| HPFS | Detailed assessments of physical activity were first obtained by questionnaires in 1986. Participants were asked to report the average amount of time they spent per week on leisure-time physical activities, including walking, jogging, running, bicycling, calisthenics or use of a rowing machine, lap swimming, squash or racquetball, and tennis. They were also asked about their usual walking pace. Based on this information, weekly energy expenditure in metabolic equivalent hours (METs) was calculated. The reproducibility and validity of the physical activity questionnaire has been described elsewhere (PMID:7860180). | Information on occupational physical activity are not available and the four level CPAI was defined in accordance with the METs quartiles. Q1 was coded as inactive, Q2 as moderately inactive, Q3 as moderately active, and Q4 as active. | Individuals within Q1 were defined as inactive, and Q2-Q4 as active | Height and body weight were self-reported in the questionnaires. Self-reported weights were highly correlated with measured weight (r=0.97 in men and women) (PMID:2090285). BMI was calculated as weight in kilograms (kg) divided by height in meters squared (m^2^) |
| INTER99 | Physical activity was assessed using questionnaire data. Occupational physical activity was categorized as i) sedentary or standing ii) light but partly physically active iii) physically active iv) physically straining most of the time v) not working. Leisure-time physical activity was categorized as i) sedentary or minimal physical activity (< 2h/week) ii) light physical activity (2-4 h/week) iii) modest physical activity (> 4 h/week) iv) highly vigorous physical activity (> 4 h/week). Among occupational physical activity (five categories) i and v were collapsed into a single category. | The CPAI was computed by cross-tabulation of occupational and leisure-time physical activity, classifying an individual’s total physical activity level according to a four-level scale (inactive, moderately inactive, moderately active and active). Among leisure-time physical activity, participants with missing information were given the lowest intensity score, i.e. classified as being ‘occasionally active’. | Participants with a CPAI=1 were classified as inactive and the remaining participants were classified as active (CPAI 2-4) | Height (cm) and body weight (kg) was measured in light indoor clothes and without shoes. BMI was calculated as weight in kilograms (kg) divided by height in meters squared (m^2^). |
| INTERACT | Physical activity was assessed using the EPIC four question physical questionnaire. The first question concerned physical activity at work. The second question asked about the amount of time spent walking, cycling, gardening, do-it-yourself, physical exercise and housework. The third question asked whether any of the activities in question 2 caused sweating or faster heartbeat and, if so, for how many hours during a typical week. The fourth question asked about stair climbing. More information can be found in a validity study (12795830). | The CPAI was computed by cross-tabulation of occupational and leisure-time physical activity, classifying an individual’s total physical activity level according to a four-level scale (inactive, moderately inactive, moderately active and active). Among leisure-time physical activity, participants with missing information were given the lowest intensity score, i.e. classified as being ‘occasionally active’. | Participants with a CPAI=1 were classified as inactive and the remaining participants were classified as active (CPAI 2-4) | Height (cm) and body weight (kg) was measured in light indoor clothes and without shoes. BMI was calculated as weight in kilograms (kg) divided by height in meters squared (m^2^). |
| MDC | Information on both physical activity at work and during leisure-time was obtained from the baseline questionnaire on lifestyle and socioeconomic status, which has not been objectively validated. A variable for total physical activity was constructed to obtain a variable comparable to the Cambridge physical activity index. The variable was based on a categorical variable for physical activity intensity at work (very light, light or medium heavy, heavy, very heavy) and a continuous variable for leisure-time physical activity (score for leisure-time physical activity based on minutes per week of different activities multiplied by intensity factors). The leisure-time physical activity variable was first divided into four categories based on approximately equal quartiles of the score (0-6000, 6000-12000, 12000-18000, >18000). | The categorical variables for physical activity at work and during leisure-time were then cross-tabulated in a manner compatible with the CPAI variable to construct four new groups: i) inactive (very light physical activity, score 0-6000), ii) moderately inactive (very light work, score 6000-12000 or light-medium heavy work, score 0-6000), iii) moderately active (very light work, score 12000-18000 or light-medium heavy work, score 6000-12000 or heavy work, score 0-6000) and iv) active (light-medium heavy work, score 12000-18000 or heavy work, score 6000-12000 or heavy work, score 12000-18000 or all combinations with very heavy work or all combinations with score>18000. | Participants with a CPAI=1 were classified as inactive and the remaining participants were classified as active (CPAI 2-4) | Height and weight were measured using wall-mounted stadiometers and calibrated balance-beam scales, respectively. BMI was calculated as weight in kilograms (kg) divided by height in meters squared (m^2^). |
| METSIM | Occupational physical activity was categorized as 1) light sedentary work; 2) sedentary work; 3) light standing work or light mobile (physical) work; 4) light or medium-heavy mobile (physical) work; 5) demanding physical work; 6) very demanding physical work; 7) retired, unemployed, etc. (missing information). Categories 1) and 2) were merged, as well as categories 5) and 6) Retired or unemployed participants were excluded from the analysis. Leisure-time physical activity was categorized as 1) a little or none, 2) physical exercise in context of other hobbies or physical exercise occasionally, 3) physical exercise regularly <=2 times a week at least 30min at a time, 4) physical exercise regularly >=3 times a week at least 30min at a time. | The CPAI was computed by cross-tabulation of occupational and leisure time physical activity, classifying an individual’s total physical activity level according to a four-level scale (inactive, moderately inactive, moderately active and active). | Binary PA was defines as the lowest quintile (approximate) of PA versus the remaining four quintiles. | Body mass index (BMI) was calculated as weight (kg) divided by height squared (m^2^). Height and weight were measured to the nearest 0.5 cm and 0.1 kg, respectively. |
| NHS | Detailed assessments of physical activity were first obtained by questionnaires in 1986. Participants were asked to report the average amount of time they spent per week on leisure-time physical activities, including walking, jogging, running, bicycling, calisthenics or use of a rowing machine, lap swimming, squash or racquetball, and tennis. They were also asked about their usual walking pace. Based on this information, weekly energy expenditure in metabolic equivalent hours (METs) was calculated. The reproducibility and validity of the physical activity questionnaire has been described elsewhere. (PMID:7860180) | Information on occupational physical activity are not available and the four level CPAI were defined in accordance with the METs quartiles.Q1 was coded as inactive, Q2 as moderately inactive, Q3 as moderately active, and Q4 as active. | Individuals within Q1 were defined as inactive, and Q2-Q4 as active | Height and body weight were self-reported in the questionnaires. Self-reported weights were highly correlated with measured weight (r=0.97 in men and women). (PMID:2090285) BMI was calculated as weight in kilograms (kg) divided by height in meters squared (m^2^) |
| TWINGENE (Q2000) | Physical activity was assessed by the following question in Screening Across the Lifespan Twin Study questionnaire: Of these 7 alternatives, which fits your annual exercise pattern? 1) Almost never exercise; 2) Much less exercise than average; 3) Less exercise than average; 4) Average amount of exercise; 5)More exercise than average; 6) Much more exercise than average; 7) Maximum amount of exercise. | Not Coded | PA binary was coded in the following way: answers 0,1,2 = inactive, answers 3,4,5,6 = active. | Height and weight were measured using wall-mounted stadiometers and calibrated balance-beam scales, respectively. BMI was calculated as weight in kilograms (kg) divided by height in meters squared (m^2^) |
| TWINGENE (Q1973) | In TwinGene study, occupational physical activity was categorized as: 1) Primarily sedentary work; 2) 1 & 3; 3) Standing and walking, but no other physical act. 4) 3 & 5; 5) Standing, walking lifting, carrying; 6) 5 & 7; 7) Physically strenuous work. Leisure time physical activity was categorized as: 1)Almost no exercise;  2) Hardly any exercise; 3) Very little exercise; 4) Little exercise; 5) Between little and much exercise; 6) Much exercise; 7) Very much exercise. Both questions were assessed with questionnaire. Among occupational physical activity we recoded the answers in: answers 1 and 2 = 'sitting', 3 and 4= ' Light', 5 and 6='Active', 7='very strenuous'. Among leisure time physical activity we recoded the answers in: answers 1 and 2 = 'Never', 3 and 4 = '1-2 / week', 5 = '2-3 / weeks', 6 and 7= '> 2-3 / weeks'. Participants with missing information in at least one of the two questions were excluded. | The Cambridge Physical Activity Index (CPAI) was computed by cross-tabulation of occupational and leisure time physical activity, classifying an individual’s total physical activity level according to a four-level scale (inactive, moderately inactive, moderately active and active). | Not Coded | Height and weight were self-reported in the same questionnaire. |
| WGHS | In the Women's Genome Health Study, leisure time physical activity was assessed by self-reported questionnaire at enrollment. Participants were asked: "How often do you engage in strenuous (aerobic) physical activity (e.g., swimming, aerobics, cycling, running)?" and chose from the following: "Rarely/never", "Less than once/week", "Once/week","2-3 times/week","4-6 times/week", and "Daily". | The CPAI was computed by recoding the 6 categories into a 4-level scale: inactive ("Rarely/never"), moderately inactive ("Less than once/week" and "Once/week"), moderately active ("2-3 times/week"), and active ("4-6 times/week" and "Daily"). | Participants with a CPAI=1 were classified as inactive and the remaining participants were classified as active (CPAI 2-4) | Height and weight were self-reported. BMI was calculated by dividing weight in kilograms by the square of height in meters. |
