# Supplementary material for: Gene × Physical Activity Interactions in Obesity: Combined Analysis of 111,421 Individuals of European Ancestry
Source: PLoS Genet. 2013 Jul 25;9(7):e1003607. doi: 10.1371/journal.pgen.1003607 (PMC3723486; doi:10.1371/journal.pgen.1003607)
Supplement: Table S8 — Genotyping methods and SNP quality control. (DOC) [file pgen.1003607.s012.doc]

**Table S8.** Genotyping methods and SNP quality control.

| **Cohort** | **Genotyping Methods** | **Nearest Gene** | **Lead SNP** | **Proxy SNP** | **r^2^ with lead SNP** | **Effect (other) allele** | | **Effect allele frequency** | **p-HWE** |
| --- | --- | --- | --- | --- | --- | --- | --- | --- | --- |
| **FENLAND** | Genotyping platform:  *Affymetrix GeneChip Human mapping 500K. Metabochip custom Illumina iSelect array* | *FTO* | rs1121980 | rs1421085 | 0.91 | C | (T) | 0.41 | N/A |
|  |  | *SH2B1* | rs7498665 | rs7359397 | 0.97 | T | (C) | 0.40 | N/A |
|  |  | *SEC16B* | rs10913469 | rs543874 | 0.96 | G | (A) | 0.21 | N/A |
|  |  | *MTCH2* | rs10838738 | rs3817334 | 0.84 | T | (C) | 0.41 | N/A |
|  | Number of imputed SNPs:  *10* | *MC4R* | rs17782313 | rs571312 | 0.96 | A | (C) | 0.25 | N/A |
|  |  | *NEGR1* | rs3101336 | rs2815752 | 0.96 | A | (G) | 0.60 | N/A |
|  |  | *TMEM18* | rs6548238 | rs2867125 | 1.0 | C | (T) | 0.82 | N/A |
|  |  | *GNPDA2* | rs10938397 | - | - | G | (A) | 0.43 | N/A |
|  | Imputation method:  *HapMap Build 36* | *BDNF* | rs925946 | rs1519480 | 1.0 | C | (T) | 0.31 | N/A |
|  |  | *KCTD15* | rs368794 | rs11084753 | 0.84 | G | (A) | 0.67 | N/A |
|  |  | *ETV5* | rs7647305 | - | - | C | (T) | 0.79 | N/A |
|  |  | *FAIM2* | rs7132908 | rs7138803 | 0.89 | A | (G) | 0.36 | N/A |
| **GLACIER** | Genotyping platform:  *OpenArray SNP Genotyping System* | *FTO* | rs1121980 | rs9939609 | 0.81 | A | (T) | 0.26 | 0.01 |
|  |  | *SH2B1* | rs7498665 | - | - | G | (A) | 0.41 | 0.66 |
|  |  | *SEC16B* | rs10913469 | - | - | G | (A) | 0.20 | 0.17 |
|  |  | *MTCH2* | rs10838738 | rs4752856 | 1.0 | T | (C) | 0.38 | 0.11 |
|  | Number of imputed SNPs:  *None* | *MC4R* | rs17782313 | - | - | C | (T) | 0.26 | 0.002 |
|  |  | *NEGR1* | rs3101336 | rs2815752 | 0.96 | A | (G) | 0.58 | 0.09 |
|  |  | *TMEM18* | rs6548238 | - | - | C | (T) | 0.82 | 0.68 |
|  |  | *GNPDA2* | rs10938397 | - | - | G | (A) | 0.37 | 0.004 |
|  | Imputation method: *N/A* | *BDNF* | rs925946 | rs4923461 | 0.19 | A | (G) | 0.80 | 0.39 |
|  |  | *KCTD15* | rs368794 | rs11084753 | 0.84 | G | (A) | 0.64 | 0.72 |
|  |  | *ETV5* | rs7647305 | - | - | C | (T) | 0.81 | 0.26 |
|  |  | *FAIM2* | rs7132908 | rs7138803 | 0.89 | A | (G) | 0.45 | 0.06 |
| **HEALTH 2006** | Genotyping platform:  *Illumina HumanCardio-Metabo BeadChip* | *FTO* | rs1121980 | - | - | T | (C) | 0.43 | 0.77 |
|  |  | *SH2B1* | rs7498665 | - | - | G | (A) | 0.46 | <0.001 |
|  |  | *SEC16B* | rs10913469 | rs543874 | 0.96 | G | (A) | 0.23 | 0.17 |
|  |  | *MTCH2* | rs10838738 | - | - | G | (A) | 0.35 | 0.14 |
|  | Number of imputed SNPs:  *None* | *MC4R* | rs17782313 | - | - | C | (T) | 0.26 | 0.79 |
|  |  | *NEGR1* | rs3101336 | - | - | G | (A) | 0.59 | 0.62 |
|  |  | *TMEM18* | rs6548238 | - | - | C | (T) | 0.83 | 0.61 |
|  |  | *GNPDA2* | rs10938397 | - | - | G | (A) | 0.41 | 0.38 |
|  | Imputation method: *N/A* | *BDNF* | rs925946 | - | - | T | (G) | 0.31 | 0.45 |
|  |  | *KCTD15* | rs368794 | - | - | A | (T) | 0.66 | 0.11 |
|  |  | *ETV5* | rs7647305 | - | - | C | (T) | 0.80 | 1.00 |
|  |  | *FAIM2* | rs7132908 | rs7132908 | 0.89 | A | (G) | 0.39 | 0.44 |
| **HPFS** | Genotyping platform:  *Illumina 550k. Affymetrix 6.0. Illumina 610Q. Illumina 660. Omni Express* | *FTO* | rs1121980 | - | - | A | (G) | 0.46 | 0.55 |
|  |  | *SH2B1* | rs7498665 | - | - | G | (A) | 0.37 | 0.81 |
|  |  | *SEC16B* | rs10913469 | - | - | G | (A) | 0.19 | 0.71 |
|  |  | *MTCH2* | rs10838738 | - | - | G | (A) | 0.36 | 0.89 |
|  | Number of imputed SNPs:  *4* | *MC4R* | rs17782313 | - | - | C | (T) | 0.24 | 0.03 |
|  |  | *NEGR1* | rs3101336 | - | - | C | (T) | 0.64 | 0.58 |
|  |  | *TMEM18* | rs6548238 | - | - | C | (T) | 0.81 | 0.20 |
|  |  | *GNPDA2* | rs10938397 | - | - | G | (A) | 0.43 | 0.11 |
|  | Imputation method: *MACH 1.0* | *BDNF* | rs925946 | - | - | T | (G) | 0.28 | 0.01 |
|  |  | *KCTD15* | rs368794 | - | - | A | (T) | 0.65 | 0.93 |
|  |  | *ETV5* | rs7647305 | - | - | C | (T) | 0.79 | 0.11 |
|  |  | *FAIM2* | rs7132908 | - | - | T | (C) | 0.41 | 0.31 |
| **INTER99** | Genotyping platform:  *Illumina HumanCardio-Metabo BeadChip* | *FTO* | rs1121980 | - | - | T | (C) | 0.44 | 1.00 |
|  |  | *SH2B1* | rs7498665 | - | - | G | (A) | 0.41 | 0.33 |
|  |  | *SEC16B* | rs10913469 | rs543874 | 0.96 | G | (A) | 0.21 | 0.45 |
|  |  | *MTCH2* | rs10838738 | - | - | G | (A) | 0.35 | 0.81 |
|  | Number of imputed SNPs:  *None* | *MC4R* | rs17782313 | - | - | C | (T) | 0.25 | 0.77 |
|  |  | *NEGR1* | rs3101336 | - | - | G | (A) | 0.59 | 0.23 |
|  |  | *TMEM18* | rs6548238 | - | - | C | (T) | 0.83 | 0.38 |
|  |  | *GNPDA2* | rs10938397 | - | - | G | (A) | 0.41 | 0.52 |
|  | Imputation method: *N/A* | *BDNF* | rs925946 | - | - | T | (G) | 0.31 | 0.17 |
|  |  | *KCTD15* | rs368794 | - | - | A | (T) | 0.66 | 0.12 |
|  |  | *ETV5* | rs7647305 | - | - | C | (T) | 0.80 | 0.39 |
|  |  | *FAIM2* | rs7132908 | rs7138803 | 0.89 | A | (G) | 0.40 | 0.04 |
| **INTERACT** | Genotyping platform:  *Illumina 660Quad and Illumina CardioMetabochip* | *FTO* | rs1121980 | - | - | A | (G) | 0.44 | 0.94 |
|  |  | *SH2B1* | rs7498665 | - | - | G | (A) | 0.37 | 0.10 |
|  |  | *SEC16B* | rs10913469 | rs543874 | 0.96 | G | (A) | 0.17 | 0.46 |
|  |  | *MTCH2* | rs10838738 | - | - | G | (A) | 0.35 | 0.69 |
|  | Number of imputed SNPs:  *None* | *MC4R* | rs17782313 | - | - | C | (T) | 0.24 | 0.38 |
|  |  | *NEGR1* | rs3101336 | - | - | C | (T) | 0.63 | 0.39 |
|  |  | *TMEM18* | rs6548238 | - | - | C | (T) | 0.83 | 0.87 |
|  |  | *GNPDA2* | rs10938397 | - | - | G | (A) | 0.43 | 0.89 |
|  | Imputation method: *N/A* | *BDNF* | rs925946 | - | - | A | (T) | 0.66 | 0.07 |
|  |  | *KCTD15* | rs368794 | - | - | T | (G) | 0.28 | 0.39 |
|  |  | *ETV5* | rs7647305 | - | - | C | (T) | 0.8 | 0.92 |
|  |  | *FAIM2* | rs7132908 | rs7138803 | 0.89 | A | (G) | 0.39 | 0.44 |
| **MDC** | Genotyping platform:  *Sequenom iPLEX platform,* *TaqMan allelic discrimination and KASPar allelic discrimination method* | *FTO* | rs1121980 | rs9939609 | 0.81 | A | (T) | 0.41 | 0.52 |
|  |  | *SH2B1* | rs7498665 | - | - | G | (A) | 0.41 | 0.91 |
|  |  | *SEC16B* | rs10913469 | - | - | C | (T) | 0.21 | 0.20 |
|  |  | *MTCH2* | rs10838738 | - | - | G | (A) | 0.35 | 0.12 |
|  | Number of imputed SNPs:  *None* | *MC4R* | rs17782313 | - | - | C | (T) | 0.23 | 0.34 |
|  |  | *NEGR1* | rs3101336 | rs2815752 | 0.96 | T | (C) | 0.58 | 0.97 |
|  |  | *TMEM18* | rs6548238 | - | - | C | (T) | 0.84 | 0.55 |
|  |  | *GNPDA2* | rs10938397 | - | - | G | (A) | 0.40 | 0.94 |
|  | Imputation method: *N/A* | *BDNF* | rs925946 | rs4923461 | 0.19 | A | (G) | 0.80 | 0.06 |
|  |  | *KCTD15* | rs368794 | rs29941 | 0.47 | C | (T) | 0.66 | 0.16 |
|  |  | *ETV5* | rs7647305 | - | - | C | (T) | 0.82 | 0.01 |
|  |  | *FAIM2* | rs7132908 | rs7138803 | 0.89 | A | (G) | 0.41 | 0.59 |
| **METSIM** | Genotyping platform:  *Illumina* | *FTO* | rs1121980 | - | - | A | (G) | 0.65 | 0.98 |
|  |  | *SH2B1* | rs7498665 | - | - | G | (A) | 0.15 | <0.001 |
|  |  | *SEC16B* | rs10913469 | - | - | G | (A) | 0.85 | 0.84 |
|  |  | *MTCH2* | rs10838738 | - | - | G | (A) | 0.82 | 0.96 |
|  | Number of imputed SNPs:  *None* | *MC4R* | rs17782313 | - | - | C | (T) | 0.52 | 0.42 |
|  |  | *NEGR1* | rs3101336 | rs2815752 | 0.96 | A | (G) | 0.35 | 0.59 |
|  |  | *TMEM18* | rs6548238 | - | - | C | (T) | 0.33 | 0.47 |
|  |  | *GNPDA2* | rs10938397 | - | - | G | (A) | 0.38 | 0.74 |
|  | Imputation method: *N/A* | *BDNF* | rs925946 | - | - | T | (G) | 0.16 | 0.60 |
|  |  | *KCTD15* | rs368794 | rs11084753 | 0.84 | G | (A) | 0.05 | 0.99 |
|  |  | *ETV5* | rs7647305 | - | - | C | (T) | 0.43 | 0.31 |
|  |  | *FAIM2* | rs7132908 | - | - | T | (C) | 0.66 | 0.005 |
| **NHS** | Genotyping platform: *Illumina 550k. Affy 6.0. Illumina 610Q. Illumina 660. Omni Express* | *FTO* | rs1121980 | - | - | A | (G) | 0.44 | 0.79 |
|  |  | *SH2B1* | rs7498665 | - | - | G | (A) | 0.39 | 0.19 |
|  |  | *SEC16B* | rs10913469 | - | - | G | (A) | 0.20 | 0.46 |
|  |  | *MTCH2* | rs10838738 | - | - | G | (A) | 0.36 | 0.69 |
|  | Number of imputed SNPs:  *4* | *MC4R* | rs17782313 | - | - | C | (T) | 0.24 | 0.86 |
|  |  | *NEGR1* | rs3101336 | - | - | C | (T) | 0.63 | 0.65 |
|  |  | *TMEM18* | rs6548238 | - | - | C | (T) | 0.83 | 0.01 |
|  |  | *GNPDA2* | rs10938397 | - | - | G | (A) | 0.44 | 0.28 |
|  | Imputation method: *MACH 1.0* | *BDNF* | rs925946 | - | - | T | (G) | 0.29 | 0.11 |
|  |  | *KCTD15* | rs368794 | - | - | A | (T) | 0.67 | 0.05 |
|  |  | *ETV5* | rs7647305 | - | - | C | (T) | 0.79 | 0.21 |
|  |  | *FAIM2* | rs7132908 | - | - | T | (C) | 0.40 | 0.30 |
| **TWINGENE (Q2000)** | Genotyping platform:  *Illumina OmniExpress bead chip* | *FTO* | rs1121980 | - | - | C | (G) | 0.81 | N/A |
|  |  | *SH2B1* | rs7498665 | - | - | G | (C) | 0.41 | N/A |
|  |  | *SEC16B* | rs10913469 | - | - | G | (C) | 0.22 | N/A |
|  |  | *MTCH2* | rs10838738 | - | - | A | (T) | 0.68 | N/A |
|  | Number of imputed SNPs:  *6* | *MC4R* | rs17782313 | - | - | T | (A) | 0.42 | N/A |
|  |  | *NEGR1* | rs3101336 | - | - | G | (C) | 0.36 | N/A |
|  |  | *TMEM18* | rs6548238 | - | - | G | (C) | 0.40 | N/A |
|  |  | *GNPDA2* | rs10938397 | - | - | C | (G) | 0.25 | N/A |
|  | Imputation method: *IMPUTE V2. HapMap 2 b36* | *BDNF* | rs925946 | - | - | C | (G) | 0.58 | N/A |
|  |  | *KCTD15* | rs368794 | - | - | T | (A) | 0.34 | N/A |
|  |  | *ETV5* | rs7647305 | - | - | A | (T) | 0.43 | N/A |
|  |  | *FAIM2* | rs7132908 | - | - | C | (G) | 0.85 | N/A |
| **WGHS** | Genotyping platform:  *Illumina HumanHap300 Duo+* | *FTO* | rs1121980 | - | - | A | (G) | 0.42 | N/A |
|  |  | *SH2B1* | rs7498665 | - | - | G | (A) | 0.39 | N/A |
|  |  | *SEC16B* | rs10913469 | - | - | C | (T) | 0.20 | N/A |
|  |  | *MTCH2* | rs10838738 | - | - | G | (A) | 0.36 | N/A |
|  | Number of imputed SNPs:  *12* | *MC4R* | rs17782313 | - | - | C | (T) | 0.24 | N/A |
|  |  | *NEGR1* | rs3101336 | - | - | C | (T) | 0.62 | N/A |
|  |  | *TMEM18* | rs6548238 | - | - | C | (T) | 0.84 | N/A |
|  |  | *GNPDA2* | rs10938397 | - | - | G | (A) | 0.43 | N/A |
|  | Imputation method:  *N/A* | *BDNF* | rs925946 | - | - | T | (G) | 0.30 | N/A |
|  |  | *KCTD15* | rs368794 | - | - | A | (T) | 0.34 | N/A |
|  |  | *ETV5* | rs7647305 | - | - | C | (T) | 0.78 | N/A |
|  |  | *FAIM2* | rs7132908 | - | - | A | (G) | 0.41 | N/A |
